# Applications of interpretable deep learning in neuroimaging: A comprehensive review

**DOI:** 10.1162/imag_a_00214

**Published:** 2024-07-12

**Authors:** Lindsay Munroe, Mariana da Silva, Faezeh Heidari, Irina Grigorescu, Simon Dahan, Emma C. Robinson, Maria Deprez, Po-Wah So

**Affiliations:** Department of Neuroimaging, King’s College London, London, United Kingdom; School of Biomedical Engineering and Imaging Sciences, King’s College London, London, United Kingdom; Institute of Clinical Medicine, University of Eastern Finland, Kuopio, Finland

**Keywords:** interpretable deep learning, explainable AI, neuroimaging, intrinsic interpretability

## Abstract

Clinical adoption of deep learning models has been hindered, in part, because the “black-box” nature of neural networks leads to concerns regarding their trustworthiness and reliability. These concerns are particularly relevant in the field of neuroimaging due to the complex brain phenotypes and inter-subject heterogeneity often encountered. The challenge can be addressed by interpretable deep learning (iDL) methods that enable the visualisation and interpretation of the inner workings of deep learning models. This study systematically reviewed the literature on neuroimaging applications of iDL methods and critically analysed how iDL explanation properties were evaluated. Seventy-five studies were included, and ten categories of iDL methods were identified. We also reviewed five properties of iDL explanations that were analysed in the included studies: biological validity, robustness, continuity, selectivity, and downstream task performance. We found that the most popular iDL approaches used in the literature may be sub-optimal for neuroimaging data, and we discussed possible future directions for the field.

## 1 Introduction

Traditionally, analysis and interpretation of neuroimaging data requires specialised expertise, is often laborious, and is subject to inter-observer variability. Therefore, deep learning (DL) has become a popular tool in neuroimaging in recent years, driven by the rise in computer processing power as well as increased access to large medical imaging datasets and the success of novel model architectures. In neuroimaging, DL has been applied to segmentation (H. Chen et al., 2018;de Brebisson & Montana, 2015;Milletari et al., 2017), super-resolution (J. Kang et al., 2015;Xu et al., 2020), image synthesis (Gong et al., 2018;Kwon et al., 2019;Shin et al., 2018) and classification (Böhle et al., 2019;M. Liu et al., 2018), among other applications. Despite the success of DL for analysing and interpreting neuroimaging data, adoption remains limited partly because DL models are often opaque and considered to be “black boxes”. In other words, the internal workings of DL models are not comprehensible to humans, which leads to concerns regarding their reliability and trustworthiness. Indeed, such “black box” models do not satisfy European General Data Protection Regulation (GDPR) legal requirements to provide “information about the logic involved” (Hacker et al., 2020).

### 1.1 Advantages of interpretable deep learning

Interpretable deep learning (iDL) has been proposed to address the opacity problem of DL models, for example, by producing explanations that highlight brain regions that are most relevant for the model predictions. iDL methods can support the translation of DL to the clinic by providing healthcare practitioners with explanations to verify predictions and communicate with patients. Additionally, deep learning practitioners can leverage iDL to debug their models and identify cases where a model makes the right decision for the wrong reason (Lapuschkin et al., 2019). iDL methods can also be employed to test scientific hypotheses, such as identifying brain regions involved in disease pathogenesis.

### 1.2 Evaluation of iDL explanations

A challenging aspect of iDL is assessing the quality of explanations because such ground truths are typically unavailable. While experts such as clinicians, pathologists, or imaging scientists can qualitatively evaluate explanations, quantitative and automated metrics are often preferred, particularly when access to medical professionals is limited. Researchers have proposed various quantitative methods to evaluate desirable properties of iDL explanations, with a particular focus on assessing fidelity and robustness (e.g.,Adebayo et al., 2018;Hooker et al., 2019;Kindermans et al., 2019;Lapuschkin et al., 2016;Montavon et al., 2018;Samek et al., 2016;M. Yang & Kim, 2019).

Fidelity refers to the extent to which explanations reflect the inner workings of the associated deep learning model. Fidelity is usually evaluated by removing features or comparing the explanations to ground truth, if available. In computer vision, feature-removal approaches generally involve masking image regions with the highest relevance in the associated explanation, obtaining predictions for the modified images, and then measuring the change in model output or accuracy. A substantial drop in accuracy indicates that the explanations faithfully highlight image features attended to by the model. For example,Montavon et al. (2018)developed a procedure to assess fidelity in which they iteratively removed4×4patches from images with the highest relevance and plotted the number of patches removed against model output score. In another example of fidelity evaluation,Adebayo et al. (2018)randomised model parameters and data labels as two sanity checks to assess whether iDL explanations truly reflected either the model mechanisms or the relationship between image features and the label. Alternatively, explanations can be compared to ground-truth maps of image features the model is expected to attend to when making predictions. For instance, bounding box annotations for objects in natural images have been used as ground truth and the ratio of mean relevance outside versus inside the bounding box has been calculated to assess the fidelity of explanations (Lapuschkin et al., 2016).

Robustness can be described as the stability of model explanations under varying modelling conditions. For example,Montavon et al. (2018)introduced the concept of continuity, which means that an iDL method should produce similar explanations for similar input images. The evaluation of iDL methods is an active research field, and for a comprehensive review of the topic, we refer readers toAlangari et al. (2023).

### 1.3 Classification of interpretable deep learning methods

Two main categories of iDL methods exist: post-hoc and intrinsic.*Post-hoc*methods use reverse engineering to generate an explanation from a “black-box” model after training. In contrast,*intrinsic*methods incorporate interpretable components into the model architecture during the design phase. Another way to classify interpretable methods is by local versus global explanations.*Local*explanations focus on individual samples and thereby increase trust in the model outcomes, whereas*global*explanations seek to provide a deeper understanding of the mechanism by which the model works.

### 1.4 Study objectives

The objectives of this review are:

1. To systematically review iDL methods applied to neuroimaging studies.
2. To review the evaluation of iDL explanations in the studies included in this review, explicitly identifying the properties evaluated and associated quantitative metrics proposed.

To the best of our knowledge, this is the first study to systematically review both post-hoc and intrinsic iDL methods in the field of neuroimaging.

We have further sub-classified iDL methods of the two categories (Table 2). Initially, we introduce five post-hoc methods (Section 4.1) and five intrinsic methods (Section 4.2) before reviewing applications to neuroimaging for each method (Section 5). Finally, we consider how iDL explanations were evaluated across the included studies (Section 6).

## 2 Systematic Search Methodology

We identified relevant articles for this review by querying PubMed, Web of Science, Google Scholar, and arXiv using the following search terms: 1. explainable, 2. XAI, 3. interpretable, 4. explainability, 5. interpretability, 6. causal reasoning, 7. counterfactuals, 8. deep learning, 9. AI, 10. neural network, 11. machine learning, 12. brain imaging, 13. neuroimaging, and 14. neuroradiology. The search terms were combined in the logical statement (1 OR 2 OR 3 OR 4 OR 5 OR 6 OR 7) AND (8 OR 9 OR 10 OR 11) AND (12 OR 13 OR 14). Articles from 2015 were included for PubMed and Google Scholar, whereas all years were included for Web of Science and arXiV due to the small number of articles returned.

Articles were initially screened based on the article title and abstract and accepted or rejected from a full-text review based on inclusion and exclusion criteria (Table 1). Only the first 500 results from Google Scholar were screened because later results were irrelevant. Finally, we extracted the pertinent information from all accepted articles into a spreadsheet for further analysis.

**Table 1.** Inclusion and exclusion criteria.

Inclusion/exclusion criteria for article screening
**Include** …both *in-vivo* and *ex-vivo* imaging.
**Exclude** …non-human subjects.
**Include** …the following imaging modalities: structural and functional magnetic resonance imaging, computed tomography, and positron emission tomography.
**Exclude** …electroencephalogram and magnetoencephalography data.
**Exclude** …non-peer reviewed articles.
**Exclude** …non-English language articles.
**Exclude** …PhD and Masters theses.
**Exclude** …reviews, surveys, opinion articles, and books. Articles must implement at least one interpretable deep learning method.
**Exclude** …interpretable methods applied to machine-learning models other than neural networks.
**Exclude** …for quality control. For example, if the explanations could not be reasonably interpreted.

## 3 Overview

The number of articles returned was 712 for PubMed, 88 for Web of Science, 1000 for Google Scholar (the upper limit), and 189 for arXiV. After title and abstract screening, the number of accepted articles was 30 for PubMed, 26 for Web of Science, 127 for Google Scholar, and 58 for arXiV. After full-text review and removal of duplicates, and added articles after a refresh, the number of accepted articles was 75 (Fig. 2).Table 2summarises the methods and papers introduced in this review.

**Fig. 1.**
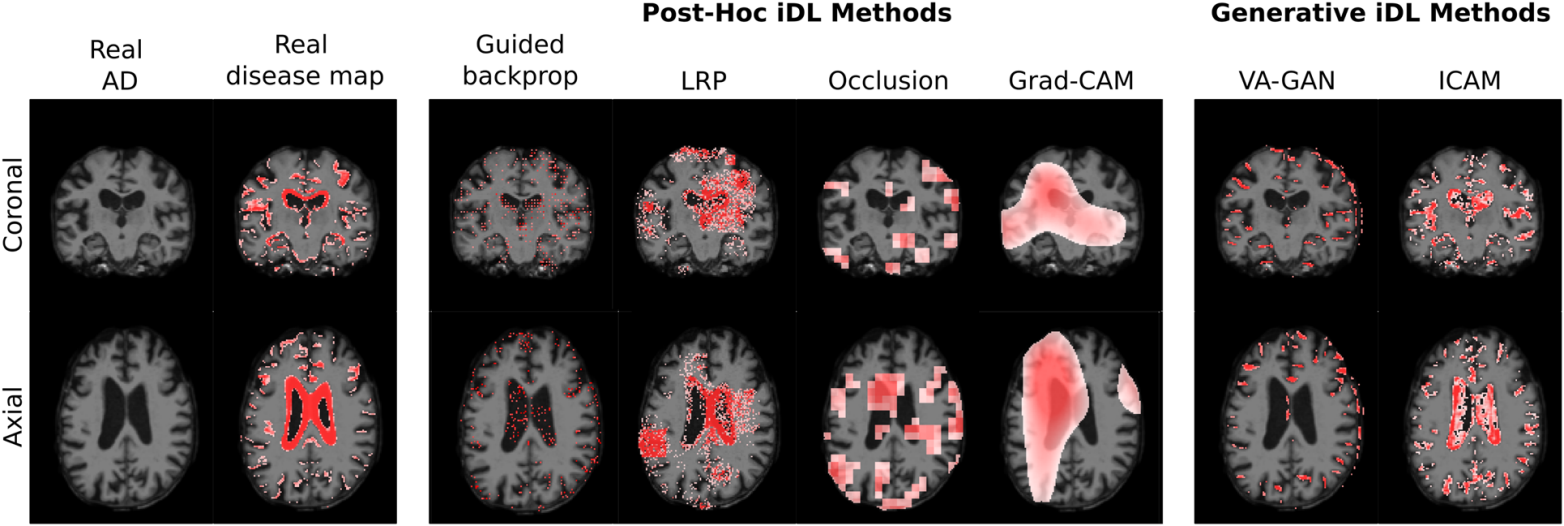
Comparison of post-hoc interpretability maps and generative interpretability methods applied to the classification of Alzheimer’s disease (AD) versus Mild cognitive impairment (MCI) in brain MRI volumes. The real disease map is the “ground-truth” shown for comparison. Figure adapted fromBass et al. (2022).

**Fig. 2.**
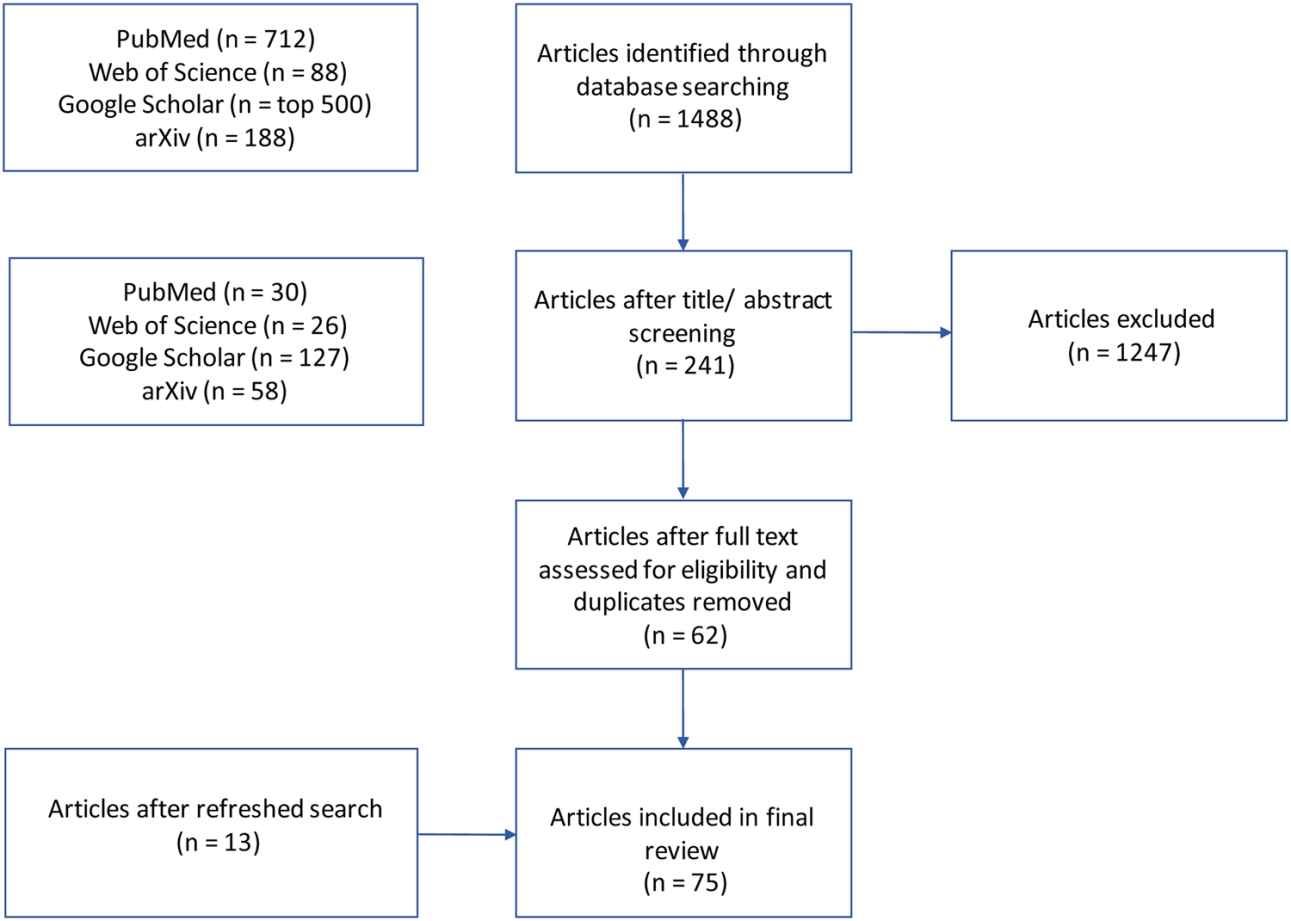
The Preferred Reporting Items for Systematic Reviews and Meta-Analyses (PRISMA) flowchart.

**Table 2.** Reviewed applications of post-hoc and intrinsic interpretable methods.

Method ▶ Application		Papers
**Post-hoc methods**	Tab. 3 **Perturbation-based methods**	
▶ Disease classification	Dhurandhar et al. (2018) , Eitel et al. (2019) , Li et al. (2018) , Y. Liu et al. (2019) , Magesh et al. (2020) , Mellema et al. (2020) , Nigri et al. (2020) , Shahamat and Abadeh (2020) , Tang et al. (2019) , Thibeau-Sutre et al. (2020) , Yan et al. (2019) , C. Yang et al. (2018)
▶ Sex classification	Kan et al. (2020)
▶ Brain age regression	Bintsi et al. (2021)
Tab. 4 **Gradient-based methods**	
▶ Disease classification	Eitel et al. (2019) , Essemlali et al. (2020) , Li et al. (2019) , Oh et al. (2019) , Q. Zhao, Adeli, Pfefferbaum, et al. (2019)
▶ Brain age regression	Levakov et al. (2020)
▶ Cognitive task decoding	Ismail et al. (2019) , McClure et al. (2023)
Tab. 5 **Backpropagation-based methods**	
▶ Disease classification	Böhle et al. (2019) , Eitel et al. (2019)
▶ Sex classification	Kan et al. (2020)
▶ Cognitive task decoding	Thomas et al. (2019)
Tab. 6 **Class activation maps**	
▶ Disease classification	Azcona et al. (2020) , Khan et al. (2019) , H. Lee et al. (2019) , Li et al. (2020) , Tang et al. (2019) , Williamson et al. (2022) , Windisch et al. (2020) , C. Yang et al. (2018) , Zhang et al. (2021)
▶ Sex classification	Gao et al. (2019) , Kan et al. (2020) , Kim and Ye (2020)
▶ Tissue segmentation	Natekar et al. (2020)
▶ Cognitive score prediction	W. Hu et al. (2021) , Qu et al. (2021)
Tab. 7 **Weight Analysis**	
▶ Disease classification	Dvornek et al. (2019) , Li et al. (2021)
▶ Tissue segmentation	Kori et al. (2020) , Natekar et al. (2020)
▶ Cognitive task decoding	Li et al. (2021)
**Intrinsic methods**	Tab. 8 **Disentangled latent spaces**	
▶ Image generation	Mouches et al. (2021) , Ouyang et al. (2022) , Q. Zhao, Adeli, Honnorat, et al. (2019) , F. Zhao et al. (2023) , Zuo et al. (2021)
▶ Disease classification	Afshar et al. (2018) , T. Wang et al. (2023)
▶ Brain age regression	D. Hu et al. (2020)
Tab. 9 **Hybrid models**	
▶ Disease classification	Abuhmed et al. (2021) , E. Kang et al. (2022) , E. Lee et al. (2019) , L. Y.-F. Liu et al. (2020) , Mohammadjafari et al. (2021) , Mulyadi et al. (2023) , Nguyen et al. (2022) , Qiang et al. (2020) , Qiu et al. (2020) , Wolf et al. (2023)
▶ Brain age regression	Hesse et al. (2023)
▶ Clinical score regression	Shimona D’Souza et al. (2020)
Tab. 10 **Generative models**	
▶ Disease classification	Bass et al. (2020) , Bass et al. (2022) , Baumgartner et al. (2018) , Lanfredi et al. (2020) , Z. Liu et al. (2021)
▶ Brain age regression	Bass et al. (2022)
▶ Tissue segmentation	Bercea et al. (2023) , Sanchez et al. (2022) , Wolleb et al. (2022)
Tab. 11 **Deep structural causal models**	
▶ Image generation	Pawlowski et al. (2020) , Rasal et al. (2022) , Reinhold et al. (2021)
Tab. 12 **Attention-based models**	
▶ Disease classification	Jin et al. (2020) , Sarraf et al. (2023) , M. Zhao et al. (2022)
▶ Tissue segmentation	Gu et al. (2020)
▶ Brain age regression	Dahan et al. (2022)

## 4 Methods

### 4.1 Post-hoc methods

Post-hoc interpretability methods, as the name suggests, analyse model decisions after a network has been trained. While some post-hoc methods are model*agnostic*, that is, they can be applied to any machine learning (ML) model, in some cases, they are only applicable to a specific family of models, such as convolutional neural networks (CNNs). Agnostic post-hoc methods can be applied to “black-box” models without requiring knowledge of the model parameters, as they generally analyse feature input and output pairs. Alternatively, post-hoc methods may require access to pre-trained model information (e.g., model weights) as for gradient-based and weight-analysis methods. The explanations computed by various post-hoc methods for a disease classification model are visualised inFig. 1.

#### 4.1.1 Perturbation-based methods

Perturbation-based methods explicitly alter the input features and measure the change in the model prediction between the original and perturbed data to discover relevant features. The most salient features for a model decision are those that produce the greatest change in the model prediction when perturbed. Perturbation-based methods mainly differ according to how they alter the input features.

Several perturbation-based methods occlude input features. For example,***Occlusion***obstructs regions of an input image in a patch-wise fashion (Zeiler & Fergus, 2014). For every patch location, the change in the model output between the original and occluded image is calculated to form a sensitivity map. For classification tasks, sensitivity is the change in predicted probabilityℙ(c)of the image belonging to a class-of-interestc, as shown inFigure 3. For regression tasks, the residual difference of the model prediction is assessed.

**Fig. 3.**
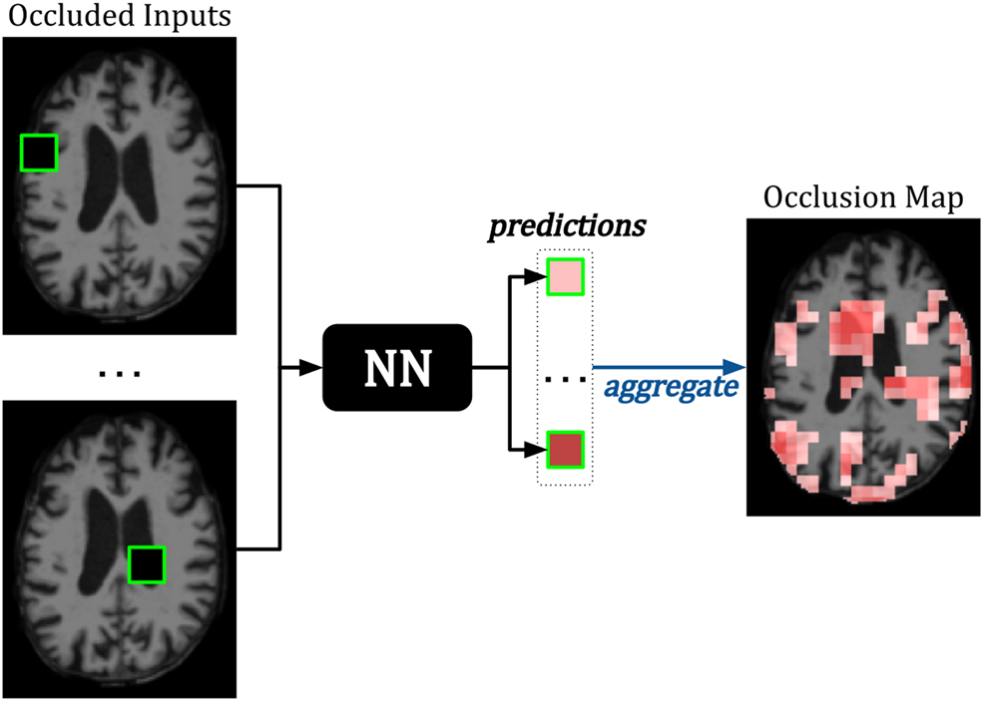
Example of***Occlusion***applied to an MRI image. In a patch-wise manner, a tile of the image is occluded, and the occluded image is fed to a neural network (NN) for prediction. The difference in predicted probability between the original and occluded image is assigned to the patch location in the occlusion map. Patches that result in the greatest change in prediction when occluded are interpreted as the most important for the model task (Zeiler & Fergus, 2014).

***Meaningful Perturbations***follows a similar approach of occluding image regions but uses gradient descent to learn the occlusion mask that obfuscates the smallest region of the image that renders the model unable to correctly classify the masked image (Fong & Vedaldi, 2017). The masking process may replace pixel values with a constant value, Gaussian noise or by blurring.

Also incorporating occlusion,***Local Interpretable Model-Agnostic Explanations (LIME)***approximates a “black-box model” locally to an inputx; then an interpretable ML model, such as a linear model, is trained to mimic the “black-box” model predictions for occluded samples ofx(Ribeiro et al., 2016). First, several perturbed images are generated from a given image$I_{0}$; a single perturbed imageIis generated by switching off a random subset of superpixels of$I_{0}$, where a superpixel is a set of neighbouring pixels with similar intensity. A sparse linear model is trained on the corresponding binary features$I^{\prime }=\left(b_{1},...,b_{n}\right)$where$b_{i}=0$if superpixel is switched off to generate imageIand$b_{i}=1$otherwise. Training labels for the linear model are the “black-box” model predictions for perturbed imagesI. The feature importance of the$i^{th}$superpixel in$I_{0}$is given by the associated linear model coefficient of$b_{i}$.

In contrast to occluding image regions, several perturbation methods swap image regions or input features with those of another subject so that the altered image still appears realistic. Such an approach was proposed in the***Swap Test***, where a reference image is selected that is from a different class to the image-of-interest (Nigri et al., 2020). For example, for an image classified as Alzheimer’s Disease (AD), the reference image is randomly selected from healthy control images. In a patch-wise manner, a patch in the reference image is replaced with the corresponding patch in the image-of-interest and the change in model output between the reference and altered reference image is computed. The process is repeated for several randomly selected reference images and averaged.

Similarly,***Permutation Feature Importance***(Fisher et al., 2019) randomly permutes values of each input feature across samples. Let$P_{\text{orig}}$be the model performance on the original data and$P_{\text{perm}}$be the model performance when featurejhas been randomly permuted; then the importance of featurejis either the ratio$P_{\text{perm}}/P_{\text{orig}}$or difference$P_{\text{perm}}-P_{\text{orig}}$. The assumption is if featurejis ignored by the model, then randomly shuffling featurejwill not influence model predictions. In contrast to previously mentioned perturbation methods, permutation feature importance is a global interpretability method.

**Advantages and disadvantages:**Perturbation-based methods have the advantage of being easy to implement and understand; they do not require a specific type of network nor access to the gradients. These methods may be applied to any “black-box” model, as they only need access to the input image and output value. However, these methods are computationally intensive and time-consuming, as inference is run for each location of the perturbation block. Another disadvantage is that perturbed images no longer belong to the training data distribution, so distribution shift may be responsible for any changes in model output rather than feature relevance (Hooker et al., 2019). Concerning*Occlusion*, this method is also sensitive to the size and the replacement intensity of the occluded patch (Fong & Vedaldi, 2017).

#### 4.1.2 Gradient-based methods

Gradient-based methods compute the partial derivative of an output from a neural network output with respect to each input feature, using the backpropagation algorithm (Rumelhart et al., 1986). The resulting gradient maps visualise how sensitive a neural network output is to small changes in input feature values, and they are also referred to as sensitivity maps.

***Vanilla Gradients***was the first gradient-based method used to compute gradient maps for a CNN trained to classify images (Simonyan et al., 2013) (seeFig. 4). Let$I_{0}$be an image withNchannels;c, a class-of-interest; and let$S_{c}\left(I\right)$be the class score output function of a trained CNN classifier. Then,*Vanilla Gradients*computes the absolute value of the partial derivative of$S_{c}\left(I\right)$with respect to each voxel in$I_{0}$. WhereN>1, the maximum value across channels is returned.

**Fig. 4.**
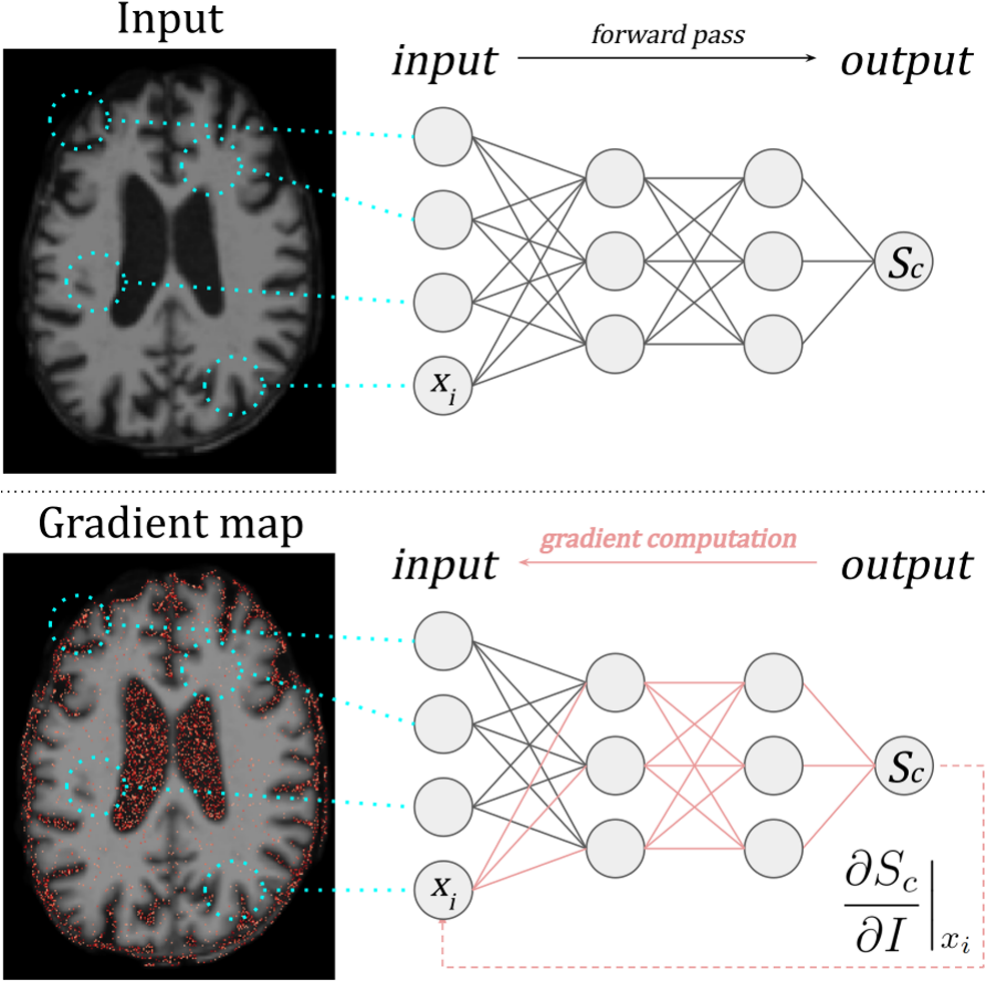
Example of***Vanilla Gradients***applied to an MRI image. Partial derivatives for each voxel with respect to the network output score$S_{c}$for classcare computed. Pixels with the largest gradients are interpreted to have the greatest influence on the model prediction (Simonyan et al., 2013).

Two main limitations of*Vanilla Gradients*exist: shattered gradients and the saturation problem. Firstly, gradient maps are often noisy because of “shattered gradients”, where similar pixel values have substantially different partial derivatives of$S_{c}$, thus producing noisy maps (Balduzzi et al., 2017). Secondly, there is the “saturation problem”. The function$S_{c}\left(I\right)$learned by a CNN is non-linear, therefore the*Vanilla Gradient*map of$I_{0}$does not interpret the behaviour of$S_{c}\left(I\right)$globally, but locally to$I_{0}$. In particular, when$S_{c}\left(I\right)$is saturated at$I_{0}$, that is, the gradient is close to zero,*Vanilla Gradients*may not reveal image features that cause$S_{c}\left(I\right)$to substantially change and switch predicted class (Shrikumar et al., 2017).

***Grad***×***Input***attempts to overcome the shattered gradients limitation through element-wise multiplication of*Vanilla Gradients*with$I_{0}$, producing visually sharper sensitivity maps than*Vanilla Gradients*(Kindermans et al., 2016).

***SmoothGrad***was also developed to address the shattered gradients limitation of*Vanilla Gradients*by adding random noise to the input image to create many noisy images, then computing the mean of the associated*Vanilla Gradients*sensitivity maps (Smilkov et al., 2017).

***Integrated Gradients***addresses the saturation problem of*Vanilla Gradients*(Sundararajan et al., 2017). Global behaviour is captured by travelling from a baseline image$I_{b}$(e.g., an image of all zeros) to the image-of-interest$I_{0}$, and samplingmimages along the path:$I_{b}+\frac{k}{m}\left(I_{0}-I_{b}\right)$for all imageskfrom 1 tom.*Integrated Gradients*then computes the mean*Vanilla Gradients*map across themimages. Notably,*Integrated Gradients*tends to highlight more relevant image features compared to*Vanilla Gradients*and*SmoothGrad*. However,*Integrated Gradients*maps may still include noisy gradients from saturated regions of$S_{c}\left(I\right)$(Miglani et al., 2020).

**Advantages and disadvantages:**Gradient-based methods are fast to run and easy to understand. However, in addition to the shattered gradients and saturation problem previously discussed, gradient maps are less able to discriminate between classes than other interpretable methods.

#### 4.1.3 Backpropagation-based methods

Backpropagation-based methods apply rules other than gradients to map the output score back to the input features to assign feature relevance. The earliest backpropagation methods for CNNs were identical to*Vanilla Gradients*aside from their treatment of the Rectified Linear Unit (ReLU) function.

Specifically,*Vanilla Gradients*back-propagates through a ReLU function by setting a gradient value to zero if the corresponding value in the forward feature map is negative. In comparison,***Guided Backpropagation***performs the same operation and also sets negative gradients to zero (Springenberg et al., 2014). Consequently,*Guided Backpropagation*only allows positive gradients, whereas*Vanilla Gradients*may produce negative gradients.

***Layer-wise Relevance Propagation (LRP)***is another popular backpropagation method, as visualised inFigure 5(Bach et al., 2015). In*LRP*, the model output score$S_{c}\left(I_{0}\right)$is redistributed backwards through the network, layer by layer, until the input image$I_{0}$is reached. Each node (or pixel) is allocated a relevance value, which is the weighted sum of relevance values of connected nodes in the neighbouring higher layer. Different LRP rules exist that each choose a different weighted sum based on the network parameters, but all follow the relevance conservation principle: relevance assigned to a node from the neighbouring higher layer is equal to the relevance passed from that node to the neighbouring earlier layer.

**Fig. 5.**
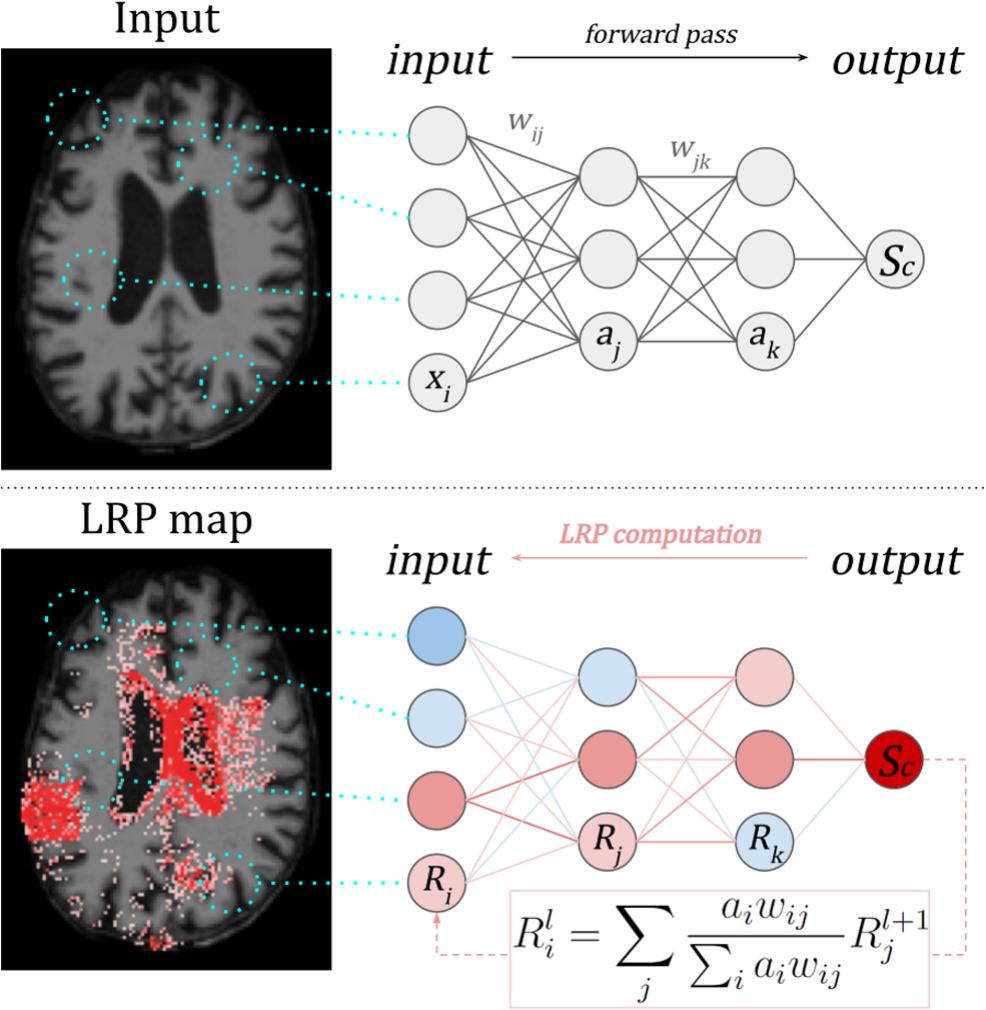
Example***Layer-wise Relevance Propagation*****(LRP)**applied to an MRI image. The network output score$S_{c}$for classcis redistributed backwards through the network according to the equation shown until the input image is reached. The pixels with the highest proportion of$S_{c}$are interpreted as having the greatest contribution to the model prediction (Bach et al., 2015).

**Advantages and disadvantages:**Analysis carried out byAdebayo et al. (2018)demonstrated*Guided Backpropagation*maps are independent of higher network layer parameters and sample labels, which is undesirable for an interpretability method. Additionally,*LRP*is sensitive to hyperparameter selection and may be difficult to tune.

#### 4.1.4 Class activation maps

***Class Activation Maps (CAM)***highlight image regions used by the final layer of a CNN to classify the input image (Zhou et al., 2016). To compute*CAM*visualisations, the final layer of the network is required to be a global average pooling (GAP) layer. In a GAP-CNN, the weighted sum of the activation maps in the final layer determines the class score$S_{c}$for each classc(Eq. 1):

$$S_{c}=\sum_{k}w_{k}^{c}\sum_{x,y}A_{x,y}^{k}=\sum_{x,y}\sum_{k}w_{k}^{c}A_{x,y}^{k}$$ (1)

where$A_{x,y}^{k}$represents the activation of nodekin the last convolutional layer of the network at pixel location(x,y), and$w_{k}^{c}$represents the importance of nodekfor the classification of classc(seeFig. 6).

**Fig. 6.**
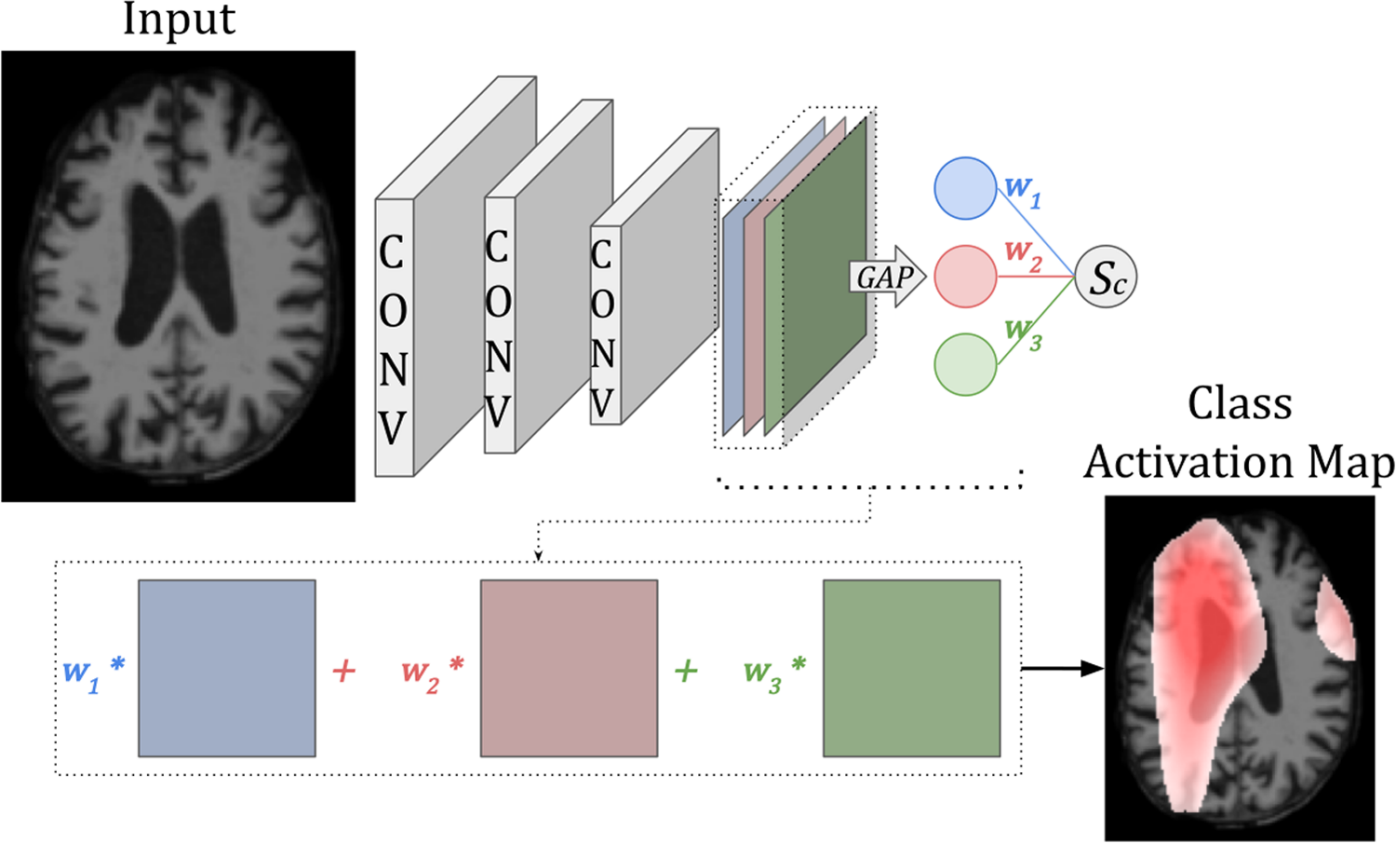
Example of a**Class Activation Map (CAM)**-based method where the activation maps of the final convolutional layer are weighted by the weights computed through the global average pooling (GAP) layer to produce a class activation map. Image adapted fromZhou et al. (2016).

Then*CAM*for classcis defined as (Eq. 2):

$$CAM^{c}=\sum_{k}w_{k}^{c}A_{x,y}^{k}$$ (2)

Hence, the sum of all elements in$CAM^{c}$is equal to the class score$S_{c}$.

***Gradient-Weighted Class Activation Maps (Grad-CAM)***extends*CAM*to all CNNs to obviate the need for a GAP layer (Selvaraju et al., 2017). In*Grad-CAM*, the weight$w_{k}^{c}$is not learned as in a*GAP-CNN*, but computed as the mean gradient of the score class$S_{c}$with respect to activation map$A_{x,y}^{k}$of a layer-of-interest (usually the last layer). Then*Grad-CAM*visualises features with positive influence only (Eq. 3):

$$\text{Grad-}CAM^{c}=ReLU\left(\sum_{k}w_{k}^{c}A_{x,y}^{k}\right)$$ (3)

Finally, the*CAM*or*Grad-CAM*heatmap is up-sampled to the original input image size and superimposed on the input image, which is why these heatmaps have a coarse resolution.

**Advantages and disadvantages:***Grad-CAM*is a popular method of interpretability, both for natural images and medical images. It is most often applied to image classification since the heatmaps are class-specific, but it can also be applied to regression and segmentation tasks.*Grad-CAM*does not require a modified CNN architecture, is not computationally intensive, is easy to implement, and is widely available in multiple libraries. A disadvantage of*CAM*and*Grad-CAM*is that the heatmaps are coarse (low resolution) because they are often upsampled from the last convolutional layer of a network. To improve the resolution,*Grad-CAM*has been coupled with other pixel-wise attribution methods such as*Guided Backpropagation*, known as***Guided Grad-CAM***. In*Guided Grad-CAM*, the*Grad-CAM*output is multiplied element-wise with the*Guided Backpropagation*heatmap.

#### 4.1.5 Weight analysis

An alternative approach for visualising and explaining the decisions of a network is to analyse the weights of the trained network. However, as deep neural networks learn high-level features in the hidden layers, simply visualising the raw learned features usually does not offer human-interpretable explanations (Molnar, 2020). Weight analysis methods attempt to create human-understandable explanations through clustering weights and associating clusters with human concepts.

The***Network Dissection***approach quantifies the interpretability of a CNN by evaluating the alignment between activated regions of individual hidden filters and human-labelled concepts (objects, parts, textures, colours) (Zhou et al., 2019). The process involves first defining a set of task-relevant concepts and then creating annotation masks$L_{c}\left(x\right)$for each conceptcand imagex. Next, masks$M_{k}\left(x\right)$of the top activated areas per filterkand per imagexare created by scaling the activation maps$A_{k}$to the size of the input images, and binarising them (thresholding on the top quantile level$T_{k}$of the distribution of pixel activations for filterkover all images). Finally, the accuracy of each filterkin detecting conceptcis reported as the sum of the Intersection over Union (IoU) between$M_{k}\left(x\right)$and$L_{c}\left(x\right)$across all the images in a dataset (seeFig. 7). To quantify the interpretability of a layer, the number of unique concepts aligned with filters, that is, unique detectors, are counted.

**Fig. 7.**
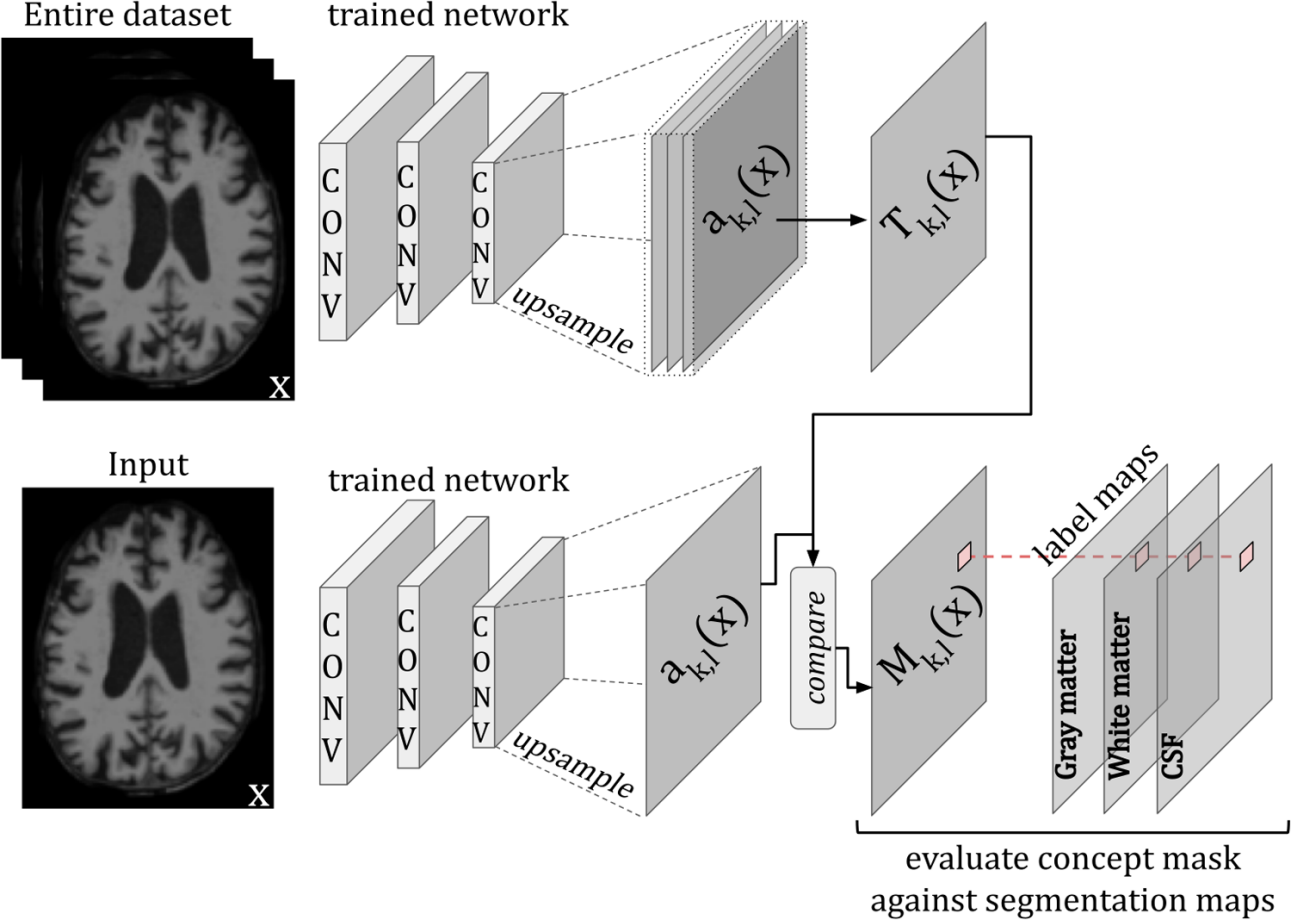
Example of a**Network Dissection**model where the activation map of individual filters in the network are analysed to identify which specific concepts they have learnt by evaluating them against segmentation maps. Image adapted fromBau et al. (2017).

A graphical representation of the concepts learned by a network to understand its behaviour was proposed (Kori et al., 2020). This***Concept Graphs***framework involves grouping similar weight vectors through hierarchical clustering in order to define concepts. Then, formed weight clusters are associated with some region in the input image by using a variation of*Grad-CAM*; the region corresponds to a human-understandable concept, for example, a tumour boundary. After the concepts have been identified, a concept graph is formed that represents the link between concepts in different layers. This is computed by intervening on the pairs of concepts and calculating the mutual information (MI) between pre-interventional and post-interventional distributions as a measure of the link between two concepts. The trails of concepts on the graph, therefore, represent the flow of information used by the network when making a prediction.

A few studies integrate***Community Detection***within a DL model for analysing functional magnetic resonance imaging (fMRI) data (Dvornek et al., 2019;Li et al., 2021). The aim of*Community Detection*in the context of neuroimaging is to discoverKnetworks of brain regions that are salient for a particular DL task. Given an fMRI connectivity matrix defined overNbrain regions, the DL model incorporates a fully connected layer with a weight matrix$W\in {\mathbb{R}}^{N\times K}$. Each value$w_{nk}\in W$may be interpreted as a membership score of brain regionnbelonging to the communityk. A clustering algorithm is then applied to the weights to assign brain regions to communities.

### 4.2 Intrinsic methods

Intrinsic interpretability refers to ML models that are explainable by design, that is, where feature representations can be understood by humans. The interpretability can be due to the simple structure of the models, such as short decision trees or sparse linear models, where network decisions can be easily followed. Alternatively, interpretability can be achieved by explicitly including interpretable modules or constraints in the model, as is required for designing iDL models. In this section, we present five categories of intrinsic interpretable methods: disentangled latent spaces, interpretable hybrid models and interpretable intermediate features, interpretable generative models, deep structural causal models, and attention mechanisms.

#### 4.2.1 Disentangled latent spaces

The latent space of a neural network is a learned representation of the input data that has usually undergone compression, such that similar input samples are transformed into representations that are close together in this space. A popular DL model is the autoencoder (AE), where an encoder learns to compress input data to a latent space, and a decoder learns to reconstruct the input data from the latent representations. An extension to the AE that enables data generation from the latent space is the variational autoencoder (VAE), where the latent space is constrained to a multivariate Gaussian distribution (Kingma & Welling, 2013). A desirable property is that the latent space is to some extent*disentangled*, meaning a single factor in the latent space corresponds to a single source of variation in the high-dimensional image space. This can be encouraged through the introduction of losses, which optimise for a subset of the latent space to encode specific semantic features in the image space. This is illustrated inFigure 8, showing a traditional versus disentangled (for subject age) latent space of a trained VAE. Note that the structure of the latent space projection in 2D space is more coherent for the disentangled space than it is for the traditional space.

**Fig. 8.**
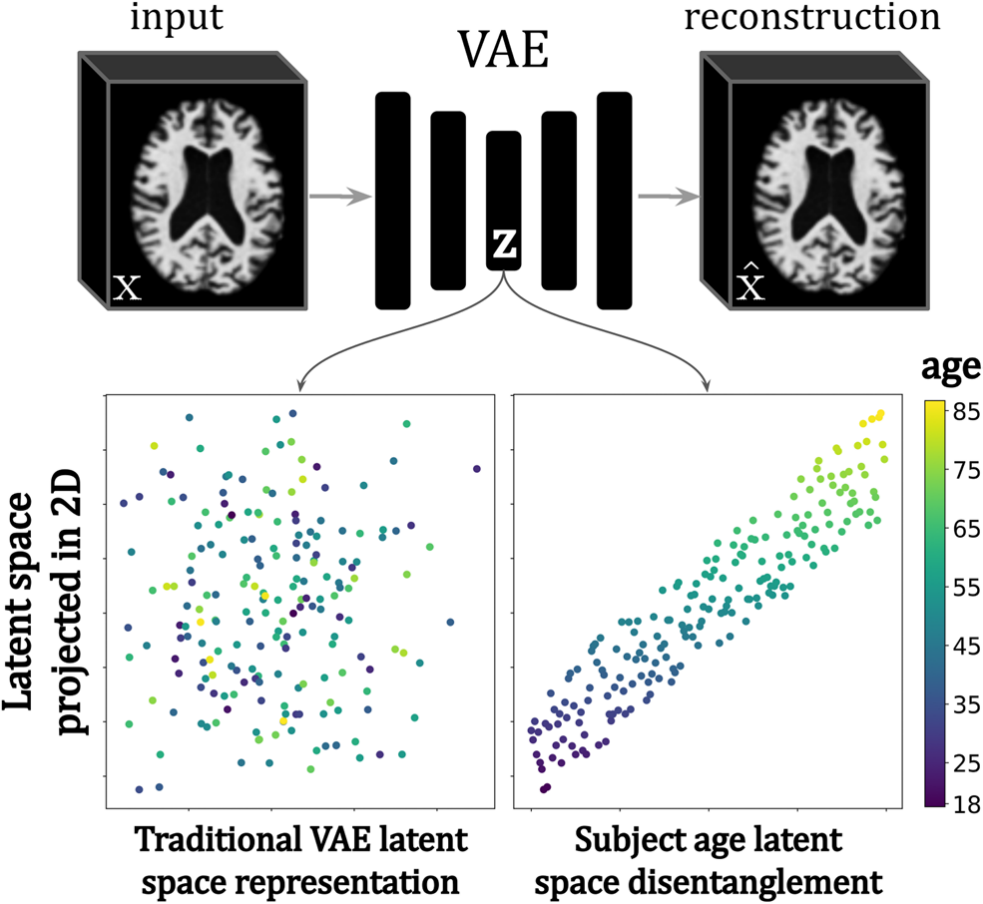
Example of a traditional versus a**disentangled latent space**of a trained VAE where age was added as a condition. The structured latent space can, therefore, be used to generate new samples for a given condition (such as age), as well as understand what type of changes occur in a given brain image with age. Image adapted fromQ. Zhao, Adeli, Honnorat, et al. (2019).

***Capsule Networks***are an alternative architecture to CNNs that learn disentangled, interpretable activation vectors (Sabour et al., 2017).*Capsule Networks*learn spatial relationships between an object and its constituent parts, which are invariant to the object viewpoint. Elements of an activation vector learn pose parameters for an associated object, such as size, orientation, texture, and hue. The$L_{2}$-norm (equal to the Euclidean distance from the origin) of an activation vector is equal to the predicted probability of the corresponding object, thus enabling classification.

**Advantages and disadvantages:**Disentangled latent representations provide some control for image generation to the end user. The user can manipulate features in the generated image in a semantically meaningful way by interpolating a disentangled factor in the latent space. One limitation of disentangling latent spaces for complex data is that the generative factors may not be inherently independent, and by constraining the latent representation to have independent representations, useful information about these dependencies can be lost (Mathieu et al., 2019). Additionally, constraining the latent space representations often comes at the expense of performance (Higgins et al., 2017). One disadvantage of ProtoPNet, in particular, is that distance maps are upsampled from the latent space to the image space, which implicitly assumes that spatial relationships in the image space are preserved in the latent space. However,Wolf et al. (2024)proved this is not necessarily the case, though efforts are being made to account for this issue (Carmichael et al., 2024;Wolf et al., 2024).

#### 4.2.2 Interpretable hybrid models and interpretable intermediate features

A*hybrid*DL model usually has two components: a neural network (NN) that learns intermediate feature representations from the input data, coupled with a model that predicts the learning task from the feature representations. The second component can be either an NN or some other ML model, referred to as NN + NN and NN + ML hybrid model, respectively.

An*interpretable hybrid model*is a hybrid model that possesses intermediate feature representations that can be understood by humans and, therefore, act as model explanations (seeFig. 9). Some researchers also compute the feature importance of the second model component and thus generate a second set of model explanations along with the intermediate features (Abuhmed et al., 2021;E. Lee et al., 2019). If a study computes feature importance as a second set of explanations, we refer to their approach as “int. features + feature importance”.

**Fig. 9.**
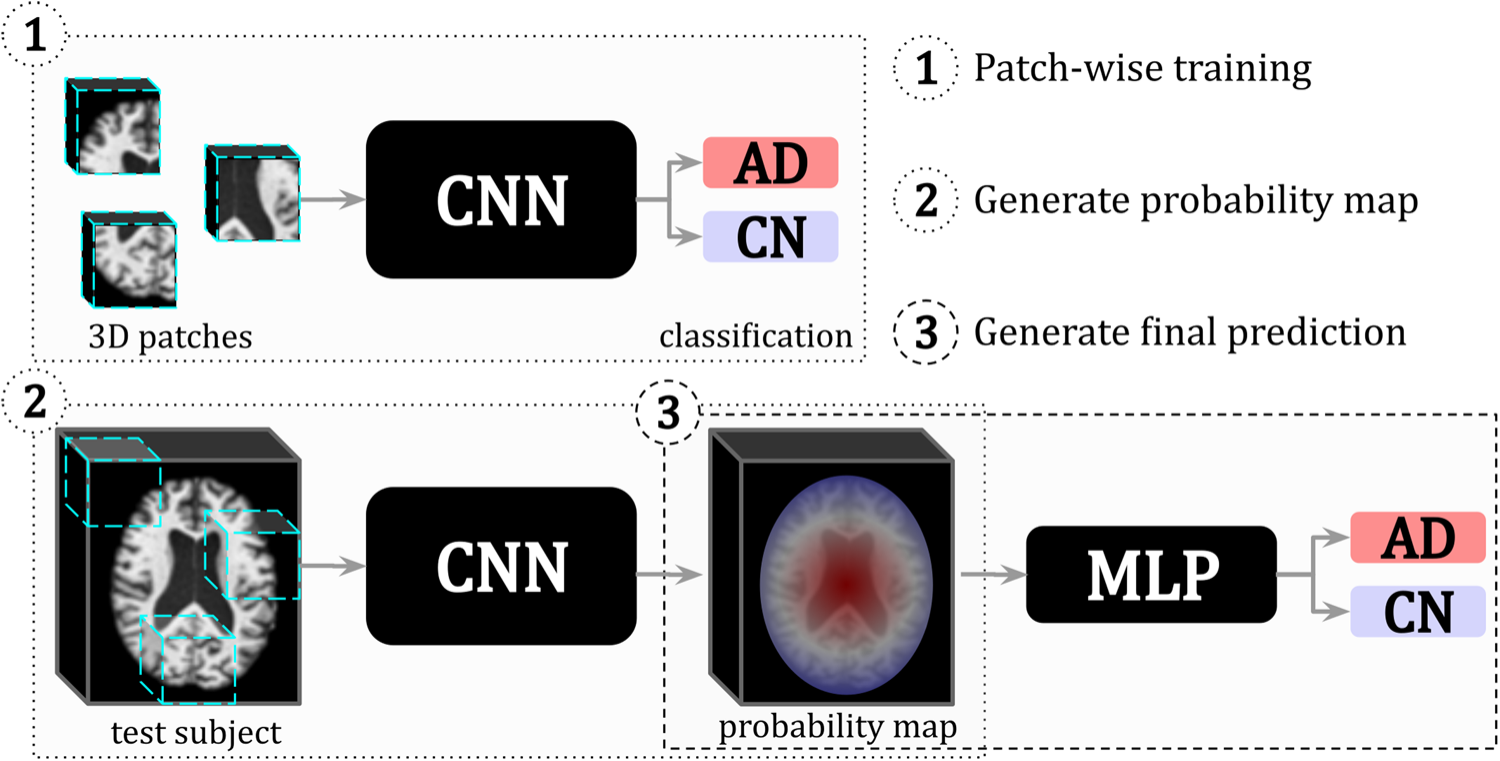
Example of an**interpretable hybrid model**where the intermediate probability map is used as the features for an multi-layer perceptron (MLP) model and acts as the model explanation. The authors of this studyQiu et al. (2020)proposed a three-step approach: first, a CNN classifier was trained to predict whether a given 3D brain magnetic resonance imaging (MRI) patch is AD or cognitively normal (CN) (1); then, the trained CNN produced a probability map (the intermediate feature) for a given test subject (2); and, finally, a multi-layer perceptron (MLP) was trained on the intermediate feature probability map to distinguish between AD and cognitively normal (CN) (3). Image adapted fromQiu et al. (2020).

One notable example of an interpretable hybrid model is the prototypical part network (ProtoPNet), which mimics human reasoning when classifying an image (C. Chen et al., 2019). The network learns a fixed number of*prototypes*for each class, where a*prototype*is a tensor in latent space that is associated with an image patch containing features typical of that class. At test time, latent features of an image are compared to each*prototype*by computing a maximum similarity score, and the similarity scores are passed through a fully connected layer to predict the image class. Several studies in our review employed*prototype*layers in their model architecture, inspired by ProtoPNet (Mohammadjafari et al., 2021;Mulyadi et al., 2023;Wolf et al., 2023).

**Advantages and disadvantages:**An advantage of interpretable hybrid models is that they may be designed so the intermediate features are suited for a particular application. For example, in a clinical setting, intermediate diagnostic features may be learned that are familiar to clinicians. However, interpretable hybrid models require careful design and may take a long time to develop.

#### 4.2.3 Interpretable generative models

Another interpretability approach is to train a generative model to generate explanations for neuroimaging tasks. The model learns to generate modifications to the input image so that the modified image appears to belong to a different class. The modifications are then used as explanations for the prediction task. For example, in binary classification, the modelfmodifies an input imagexof class0, such that$x^{\prime }=f\left(x\right)$appears to be from class1. This task is often referred to as anomaly detection or counterfactual generation, and the modifications are known as the anomaly map or disease effect map. Such an example is shown inFigure 10where a network produces the minimal additive mask needed to change an image from one class, AD in this case, to another, for example, cognitively normal (CN).

**Fig. 10.**
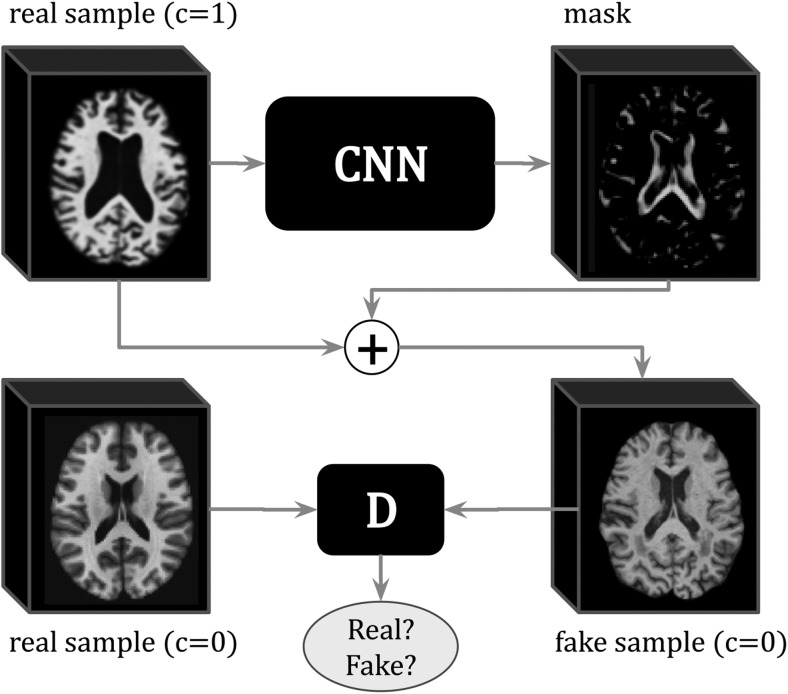
Example of an**interpretable generative model**where a generator network (shown as**“CNN”**in the figure) produces the minimum mask needed to change the class of the input sample (fromc=1toc=0). A discriminator network (**“D”**) is trained to distinguish between fake and real samples of the same class c in order to constrain the generator to produce realistic samples. Moreover, the masks can be used as explanations for the class discriminative features. Image adapted fromBaumgartner et al. (2018).

**Advantages and disadvantages:**By learning to generate new images as explanations for discriminative tasks, generative methods are capable of capturing more meaningful class-discriminative features in comparison to methods that evaluate the features learned by classification networks. These generative methods also provide a framework to investigate how changing features in an image, for example, by interpolation, affects the network decision. However, generative models can be challenging to train and require high computational power, rendering these methods harder to implement.

#### 4.2.4 Deep structural causal models

**Fig. 11.**
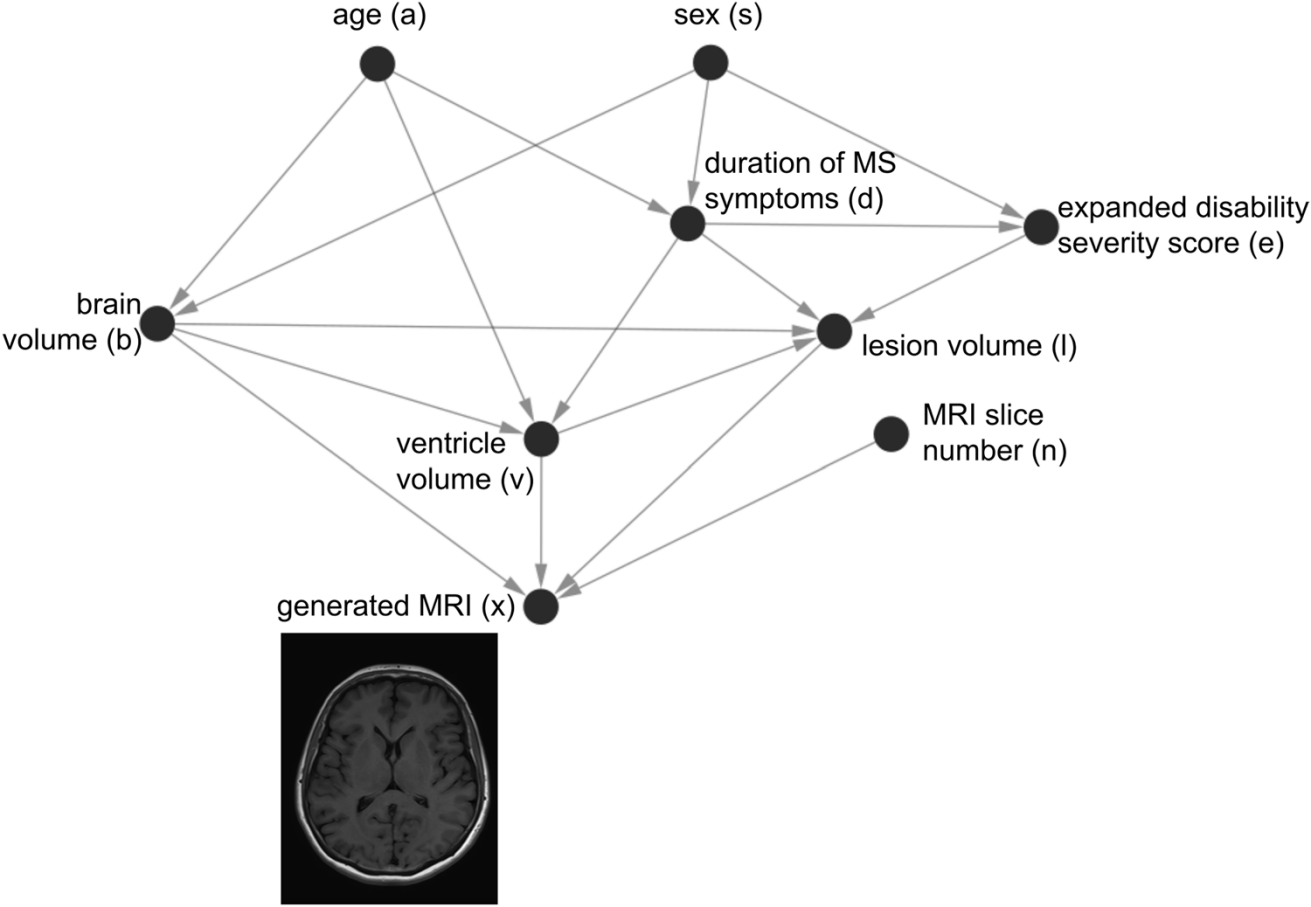
**Example of a causal graph where assumptions about the image generating mechanism are explicitly defined.**Deep structural causal models can then be learned to estimate MRI images under counterfactual scenarios. Image adapted fromReinhold et al. (2021).

Where randomised controlled trials are impossible, infeasible, or unethical, estimating causal effects is often still possible using causal inference methods. One such method is the*Structural Causal Model (SCM)*, which estimates causal effects by simulating population-level interventions (Pearl, 2009). An SCM consists of a set ofdendogenous variables$\left\{X_{1},...,X_{d}\right\}$, exogenous or noise variables$\left\{N_{1},...,N_{d}\right\}$, and structural assignments (denoted as:=):

$$X_{j}:=f_{j}\left(PA_{j},N_{j}\right),j=1,...,d$$ (4)

where$PA_{j}\subseteq \left\{X_{1},...,X_{d}\right\}\backslash \left\{X_{j}\right\}$are the parents of$X_{j}$. The joint probability distribution over the noise variables is assumed to be jointly independent. An SCM has an associated causal graphGthat visually represents our assumptions regarding how data were generated in the real world. The causal graphGis a directed acyclic graph (DAG) where all endogenous variables are represented as nodes. A directed edge$X_{i}$→$X_{j}$exists inGif$X_{j}$depends on$X_{i}$for its value. Indeed, we define$X_{i}$to be a direct cause of$X_{j}$if$X_{i}$appears in the structural assignment$f_{j}$for$X_{j}$.Figure 11is an illustration of a causal graph for Multiple Sclerosis (MS).

For an SCM, the causal effect of intervening on a variableXby setting it toais denoteddo(X=a). It is also possible to estimate*counterfactual*scenarios for specific individuals, which are hypothetical alternative outcomes to the actual outcome. We refer readers toPearl et al. (2016)andPeters et al. (2017)for a detailed overview of SCMs.

*Deep Structural Causal Models (DSCMs)*employ neural networks to learn at least one of the structural assignments in the SCM, and applying them to medical imaging data is an emerging research topic (Castro et al., 2020;Pawlowski et al., 2020;Rasal et al., 2022;Reinhold et al., 2021).Pawlowski et al. (2020)trained a*DSCM*on UK Biobank data to understand how a subject’s age (a), sex (s), brain volume (b), and ventricle volume (v) influenced their brain magnetic resonance imaging (MRI) image (img). The structural assignments were defined as (Eq. 5):

$$\begin{array}{l} b:=f_{1}\left(a,s,N_{1}\right)\\ v:=f_{2}\left(a,b,N_{2}\right)\\ img:=f_{3}\left(v,b,N_{3}\right) \end{array}$$ (5)

where$N_{1},N_{2},N_{3}$are noise variables. In this study,$f_{1}$and$f_{2}$were modelled with normalising flows, and$f_{3}$was learned using a conditional VAE where the VAE generated an estimated brain MRI. A normalising flow is a sequence of invertible transformations$g=f_{1}\circ f_{2}\circ \text{…}\circ f_{K}$that transforms a tractable distributionzinto a more complex distributionx=g(z)(we refer readers toKobyzev et al. (2020)for an introduction to normalising flows). Images were generated for a variety of counterfactual scenarios, and difference maps between the generated and original images were visually inspected for interpretation. For instance, for a 49-year-old subject, an image was generated for the counterfactualdo(age=80year-old); the generated image exhibited increased ventricle volume and reduced brain volume compared to the original image, consistent with trends in the true distribution.

**Advantages and disadvantages:**The strength of*DSCMs*is that causal mechanisms of imaging markers may control for confounders, unlike most other DL models. However, the causal graphGmust be carefully constructed from domain knowledge, and the structure ofGmay not yet be fully elucidated. Furthermore, it is impossible to obtain ground-truth data for counterfactual scenarios, so counterfactual images cannot be validated.

#### 4.2.5 Attention mechanisms

In recent years, attention in the context of deep learning has become an important area of research as it can be easily incorporated into existing neural network architectures while also improving performance (Brauwers & Frasincar, 2021;Niu et al., 2021) and providing explanations (Samek et al., 2017;Singh et al., 2020).***Attention***methods learn a heatmap over the inputs, features, or channels of the neural network, subsequently used to weight the data to emphasise key features. In the following, we discuss four main types of*attention*: channel, spatial, non-local, and self-attention, which are illustrated inFigure 12. For a more comprehensive description of DL*attention*mechanisms, we refer the reader toNiu et al. (2021)andGuo et al. (2022).

**Fig. 12.**
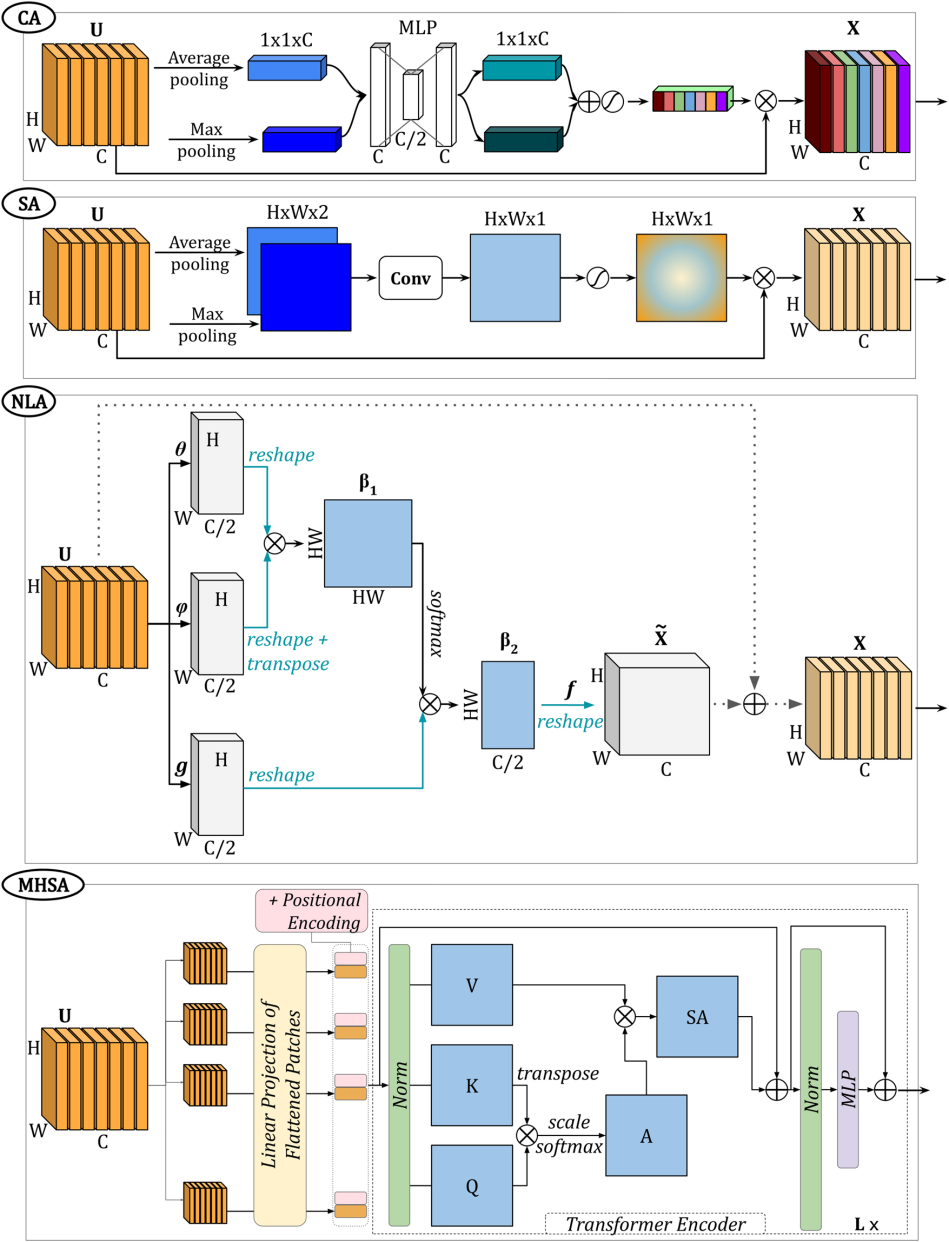
Example of**attention-based methods**showing channel attention in the first panel (**“CA”**), spatial attention in the second panel (**“SA”**), non-local attention in the third panel (**“NLA”**), and multi-head self-attention in the fourth panel (**“MHSA”**). Note that**“CA”**and**“SA”**are adapted fromWoo et al. (2018),**“NLA”**is adapted fromX. Wang et al. (2018), while the fourth panel is adapted fromDosovitskiy et al. (2020).

***Channel attention***assigns a weight to each filter in order to emphasise useful features. One of the most popular*channel attention*blocks is the*squeeze-and-excitation*block (J. Hu et al., 2018). LetUbe a feature map with dimensionsH×W×C, then the*squeeze-and-excitation*block comprises a*squeeze*function ($F_{sq}$), which performs global average pooling (Lin et al., 2013), followed by an*excitation*function ($F_{ex}$) defined as the sigmoid function (σ) applied to an multi-layer perceptron (MLP). More specifically, the*squeeze-and-excitation channel attention*$\alpha_{SE}$is defined as (Eq. 6):

αSE=Fex(Fsq(U))=σ(W2 ReLU (W1 GlobalAvgPool(U))) (6)

Here,$W_{1}\in {\mathbb{R}}^{\text{C/2}\times \text{C}}$and$W_{2}\in {\mathbb{R}}^{\text{C}\times \text{C/2}}$are the weights of the fully connected layers of the MLP. A different flavour of*channel attention*was proposed byWoo et al. (2018). Here, both global max pooling and global average pooling layers are included to generate twoC-dimensional descriptors. The final*channel attention*map$\alpha_{C}$is (Eq. 7):

αC=σ(W2 ReLU (W1 GlobalAvgPoo (U))+W2 ReLU (W1 GlobalAvgPool (U))) (7)

***Spatial attention***aims to extract important information in the image domain or across the spatial dimensions of a feature map. InWoo et al. (2018), the*spatial attention*block first performs average and max pooling operations across the channels of the inputU, generating two feature maps which are then concatenated. A convolutional layer is then applied to produce a 1-channel spatial map which, after passing through the sigmoid function, becomes the attention map ($\alpha_{S}$) (Eq. 8):

$$\alpha_{S}=\sigma \left(f\left(\left[\text{AvgPool}\left(U\right);\;\;\text{MaxPool}\left(U\right)\right]\right)\right)$$ (8)

where[⋅;⋅]represents channel-wise concatenation andfis the convolutional layer.

Oktay et al. (2018)introduced a different version of*spatial attention*with Attention U-Net, where attention gates apply convolutions to both features from the encoder and the corresponding decoder and then fuse them together to create the attention map. Moreover, instead of simply concatenating the encoder and decoder feature maps as for U-Net skip connections, the authors first scaled the encoder features with the generated*spatial attention*.

***Non-local attention***, proposed byX. Wang et al. (2018), aims to capture long-range dependencies by computing interactions between any two positions in an image or feature map. Conversely,*channel*or*spatial attention*focuses mainly on local information, that is, the pooling operation leads to loss of spatial information, while convolutional layers process neighbourhood information.

In*non-local attention*, three parallel1×1convolutional operations (θ,ϕandg) are applied on the inputU, obtaining three compressed feature maps, while a final1×1convolutional operationfrestores the initial number of channels. Introduced byLin et al. (2013), the1×1convolutions act as a channel-wise pooling operator. The non-local attention map$\alpha_{NL}$is obtained through the following operations:

$$\alpha_{NL}=\tilde{X}=f\underset{\beta_{2}\in {\mathbb{R}}^{\text{HW}\times \text{C}/2}}{\underset{︸}{\left(\text{softmax}\underset{\beta_{1}\in {\mathbb{R}}^{\text{HW}\times \text{HW}}}{\underset{︸}{\left(\theta \left(U\right)⊗\phi {\left(U\right)}^{T}\right)}}⊗g\left(U\right)\right)}}$$ (9)

whereTis the matrix transpose operation,⊗is the matrix multiplication operator, and$\tilde{X}$,$\beta_{1}$and$\beta_{2}$are shown inFigure 12. Moreover, the outputs of the convolutional layersθ,ϕandgare reshaped to allow for matrix multiplication, that is, they become 2D matrices of shapeHW×C/2. These steps are shown in the third panel ofFigure 12.

Finally,***self-attention***is a mechanism in deep learning, closely related to the concept of*non-local attention*(X. Wang et al., 2018), commonly used in natural language processing (NLP) tasks, particularly in transformer-based architectures (Vaswani et al., 2017).Dosovitskiy et al. (2020)adapted the*self-attention*model to image-based applications.

In architectures based on transformers (Dosovitskiy et al., 2020;Hatamizadeh et al., 2022) which employ*self-attention*modules, the initial step involves splitting the input data into a sequence of patches. Subsequently, these patches undergo processing via a linear projection layer and are merged with positional encodings to incorporate spatial biasing within the patch sequence (seeFig. 12). The embedded patches are then passed through a “transformer encoder” which consists of alternating layers of*multi-head self-attention*and MLP blocks, as well as residual connections and normalisation layers. More specifically, a*multi-head self-attention*block is composed of multiple parallel*self-attention*heads, which compute attention scores based on the query (Q), key (K), and value (V) representations of the input as follows:

$$SA=\text{softmax}\left(\frac{QK^{T}}{\sqrt{D_{h}}}\right)V$$ (10)

where$D_{h}$is a scaling factor.

**Advantages and disadvantages:***Attention*-based methods often add computational complexity to an existing DL model, but have a differentiable objective and are easily trainable with gradient descent. Moreover, they aim to provide a weighting for inputs or internal features to focus the network on salient characteristics. However, whether*attention*can be regarded as feature importance is an ongoing debate (Jain & Wallace, 2019;Serrano & Smith, 2019;Wiegreffe & Pinter, 2019).

## 5 Applications

### 5.1 Applications of perturbation-based methods ( Table 3 )

**Table 3.** Articles using perturbation-based interpretable methods.

Reference	Data	Modality	#Subjects	Method
**AD Classification**				
Eitel et al. (2019)	ADNI	sMRI (3D)	344	Occlusion
Y. Liu et al. (2019)	ADNI	DTI (3D)	151	Occlusion
Nigri et al. (2020)	ADNI+AIBL	sMRI (3D)	1,248	Swap Test
Shahamat and Abadeh (2020)	ADNI	sMRI (3D)	140	Optimal mask
Tang et al. (2019)	In-house	Histology (2D)	33	Occlusion
Thibeau-Sutre et al. (2020)	ADNI+ AIBL	sMRI (3D)	1,171	Meaningful Pert.
C. Yang et al. (2018)	ADNI	sMRI (3D)	103	Occlusion
**PD Classification**				
Magesh et al. (2020)	PPMI	SPECT (2D)	642	LIME
**ASD Classification**				
Dhurandhar et al. (2018)	ABIDE	fMRI (ts)	293	Optimal mask
Li et al. (2018)	Various ^†^	fMRI (3D)	225	Occlusion (global)
Mellema et al. (2020)	IMPAC	sMRI/fMRI (3D)	915	PFI
Shahamat and Abadeh (2020)	ABIDE	sMRI (3D)	1,000	Optimal mask
**SCZ Classification**				
Yan et al. (2019)	In-house	fMRI (3D)	1,100	Leave-one-IC-out
**Sex Classification**				
Kan et al. (2020)	HCP	sMRI (3D)	1,113	Meaningful Pert.
**Brain Age Regression**				
Bintsi et al. (2021)	UK Biobank	sMRI (3D)	13,750	U-Noise

Datasets: † In-house+ ABIDE.

ABIDE, Autism Brain Imaging Data Exchange; AD, Alzheimer’s Disease; ADNI, Alzheimer’s Disease Neuroimaging Initiative; AIBL, Australian Imaging Biomarker and Lifestyle Flagship Study of Ageing; ASD, Autism Spectrum Disorder; DTI, Diffusion Tensor Imaging; fMRI, functional Magnetic Resonance Imaging; HCP, Human Connectome Project; IMPAC, IMaging-PsychiAtry Challenge; LIME, local interpretable model-agnostic explanations; PD, Parkinson’s Disease; PPMI, Parkinson’s Progressive Markers Initiative database; SCZ, schizophrenia spectrum disorders; sMRI, structural Magnetic Resonance Imaging; SPECT, Single Photon Emission Computed Tomography.

#### 5.1.1 Neurodegenerative disease classification

*Occlusion*,*Swap Test*and*Meaningful Perturbations*have all been applied to AD classification networks trained on brain MRI images from the Alzheimer’s Disease Neuroimaging Initiative (ADNI) (Tahmasebi et al., 2010).Eitel et al. (2019),Y. Liu et al. (2019), andC. Yang et al. (2018)employed*Occlusion*to highlight image regions important for AD prediction. Furthermore,Rieke et al. (2018)andC. Yang et al. (2018)refined the*Occlusion*method by occluding brain regions defined by an atlas, instead of image tiles.Nigri et al. (2020)suggested*Occlusion*may be unsuitable for neuroimaging data, since an occluded patch in a brain image from a cognitively normal individual may appear similar to disease. Consequently, the authors proposed and applied*Swap Test*(Nigri et al., 2020).*Meaningful Perturbations*with a constant-valued mask was also applied for AD classification (Thibeau-Sutre et al., 2020). Furthermore,Shahamat and Abadeh (2020)employed a complementary approach to*Meaningful Perturbations*, in which minimal brain masks were learned for CNNs trained to classify AD and Autism Spectrum Disorders (ASD). A minimal brain mask*keeps*the fewest brain regions while still achieving high model accuracy, whereas the brain mask in*Meaningful Perturbations deletes*the fewest brain regions that cause a wrong prediction. In most of these AD studies, explanations contained salient regions known to be altered in AD such as the temporal lobe and hippocampus; however, the*Occlusion*map in one study could not be meaningfully interpreted because the occlusion window was too large (Eitel et al., 2019).

Another perturbation method,*LIME*, was applied to explain predictions of a VGG (Simonyan & Zisserman, 2014) model trained to predict Parkinson’s Disease (PD) on Single Photon Emission Computed Tomography (SPECT) data (Magesh et al., 2020).*LIME*explanations of CN individuals clearly delineated the putamen and caudate regions, whereas the highlighted areas in explanations for PD patients often extended beyond these regions.

#### 5.1.2 Autism spectrum disorder classification

Global perturbation-based methods have been employed to identify important features for ASD classification (Li et al., 2018;Mellema et al., 2020).*Permutation Feature Importance*was applied to a model trained on both structural (e.g., cortical volume and thickness) and functional MRI features (Mellema et al., 2020).Li et al. (2018)modified*Occlusion*to produce a global explanation for ASD classification. Specifically, after a CNN was trained to classify ASD, each atlas-based brain regionrwas occluded in all images. Let$P_{\text{orig}}^{\text{CN}}$and$P_{\text{orig}}^{\text{ASD}}$represent the distribution of predicted probabilities for all control and ASD subjects, respectively. Similarly, let$P_{\text{occ}}^{\text{CN}}$and$P_{\text{occ}}^{\text{ASD}}$represent the distributions for the corresponding images with regionroccluded. Then, utilising Jensen-Shannon Divergence (JSD), the distance between class distributions was computed and compared (Eq. 11):

$$\text{JSD}\left(P_{\text{orig}}^{\text{CN}},\;\;P_{\text{orig}}^{\text{ASD}}\right)>\text{JSD}\left(P_{\text{occ}}^{\text{CN}},\;\;P_{\text{occ}}^{\text{ASD}}\right)$$ (11)

Brain regionrwas considered important if the decrease in JSD for occluding that region was statistically significant. The assumption is if regionris important for ASD prediction, then the CNN will not separate classes as effectively when regionris removed.

Approaches that learn optimal brain masks for ASD classification have been used for models trained on the Autism Brain Imaging Data Exchange (ABIDE) (Di Martino et al., 2014) I dataset (Dhurandhar et al., 2018;Shahamat & Abadeh, 2020).Dhurandhar et al. (2018)produced the pertinent positive$\delta_{pos}$and negative$\delta_{neg}$features for a given resting-state fMRI image$I_{0}$. The former,$\delta_{pos}$, are minimally sufficient meaning the network will predict the same class for both$I_{0}$and$\delta_{pos}$. In contrast,$\delta_{neg}$must be absent for the prediction, that is, the network predicts a different class for the perturbed image$I_{0}+\delta_{neg}$compared to$I_{0}$. Similarly,Shahamat and Abadeh (2020)learned minimal brain masks for structural MRI images from ABIDE I, using the same approach they applied to the ADNI dataset, previously described inSection 5.1.1. The important brain regions identified in all these ASD studies were disparate with little overlap, although motor regions were frequently highlighted.

#### 5.1.3 Schizophrenia classification

A method analogous to*Occlusion*was applied to a recurrent neural network (RNN) trained to classify Schizophrenia (SCZ) on resting-state functional magnetic resonance imaging (rs-fMRI) data (Yan et al., 2019). Initially, the data underwent dimensionality reduction using independent component analysis (Calhoun et al., 2001), and the time series of 50 valid independent components (ICs) were retained. After RNN training, feature importance of the$i^{th}$IC was computed by replacing the$i^{th}$IC time series values with its average (essentially occluding the$i^{th}$feature), and then the change in model performance was assessed. The ICs with the greatest change in performance were considered to be the most important features for classification, which were located in the dorsal striatum and cerebellum.

#### 5.1.4 Brain age regression

The***U-noise***method, similar to*Meaningful Perturbations*, trained a U-net (Ronneberger et al., 2015) by adding maximum random noise to input images without affecting the performance of a pre-trained prediction model (Koker et al., 2021). A sensitivity map can be generated to show the image pixels that were least tolerant to the addition of noise.Bintsi et al. (2021)adapted the*U-noise*architecture to interpret a 3D ResNet (He et al., 2016) trained for brain age regression on UK Biobank (Sudlow et al., 2015)$T_{1}$-weighted MRI data. They computed an average importance map across all subjects for the test dataset. However, changes in the cerebral cortex related to aging were not well captured by the network.

#### 5.1.5 Sex classification

*Meaningful Perturbations*was compared to two alternative iDL methods for visualising model decisions for sex classification (Kan et al., 2020). The*Meaningful Perturbations*explanation highlighted regions of the frontal lobe, though the explanations were visually dissimilar between the three methods.

### 5.2 Applications of gradient-based methods ( Table 4 )

**Table 4.** Articles using gradient-based methods.

Reference	Data	Modality	#Subjects	Method
**AD classification**				
Eitel et al. (2019)	ADNI	sMRI (3D)	344	Grad × Input
Essemlali et al. (2020)	ADNI	DW-MRI (2D)	186	Vanilla Gradients
Oh et al. (2019)	ADNI	sMRI (3D)	694	Vanilla Gradients
**ASD classification**				
Li et al. (2019)	In-house	fMRI (ts)	118	Vanilla Gradients
**HIV classification**				
Q. Zhao, Adeli, Pfefferbaum, et al. (2019)	In-house	sMRI (3D)	355	Vanilla Gradients
**Brain age regression**				
Levakov et al. (2020)	Various ^†^	sMRI (3D)	10,176	SmoothGrad
**Cognitive task decoding**				
Ismail et al. (2019)	HCP	fMRI (ts)	749	Vanilla Gradients
McClure et al. (2023)	HCP	fMRI (3D)	965	Vanilla Gradients

Datasets: †= ABIDE+ ADNI+ AIBL+ IXI+ others.

ABIDE, Autism Brain Imaging Data Exchange; AD, Alzheimer’s Disease; ADNI, Alzheimer’s Disease Neuroimaging Initiative; AIBL, Australian Imaging Biomarker and Lifestyle Flagship Study of Ageing; ASD, Autism Spectrum Disorder; DW-MRI, Diffusion-Weighted Magnetic Resonance Imaging; fMRI, functional Magnetic Resonance Imaging; HCP, Human Connectome Project; HIV, Human Immunodeficiency Virus; IXI, Information Extraction from Images; sMRI, structural Magnetic Resonance Imaging; ts, time series.

#### 5.2.1 Neurodegenerative disease classification

Several studies employed*Vanilla Gradients*or*Grad*×*Input*to identify important brain regions after training a CNN on MRI data from ADNI for AD classification (Eitel et al., 2019;Essemlali et al., 2020;Oh et al., 2019;Rieke et al., 2018). All studies followed a similar approach, where class-average gradient maps were computed and then compared to explanations from other methods.Oh et al. (2019)produced*Vanilla Gradients*maps for AD classification and found they were in agreement with*Occlusion*maps.Essemlali et al. (2020)focused on connectivity between brain regions from diffusion-weighted MRI data, and found*Vanilla Gradients*highlighted relevant brain regions, unlike*Occlusion*.Eitel et al. (2019)analysed robustness across model runs for*Grad*×*Input*,*Occlusion*, and two additional methods, and concluded*Grad*×*Input*produced the least consistent explanations. To aid interpretation of*Vanilla Gradients*maps,Rieke et al. (2018)computed a quantitative relevance score by summing sensitivity within each atlas-based brain region.Eitel et al. (2019)similarly explored three summary statistics across brain regions, including mean region sensitivity, to account for differences in brain region volume. All studies determined the medial temporal lobe and/or the hippocampus as important regions for AD classification.

#### 5.2.2 Autism spectrum disorders classification

*Vanilla Gradients*has been applied to identify important features for ASD classification from task-based fMRI data, where the task was testing perception of people’s movements (biopoint task) (Li et al., 2019). A graph was constructed with each node corresponding to a specific brain region, and having an associated feature vector of 10 researcher-selected features. After training a graph neural network (GNN) and computing the gradient of the network output with respect to each feature, sensitivity maps were averaged across nodes and subjects to generate a sensitivity score per feature.

#### 5.2.3 Human immunodeficiency virus classification

*Vanilla Gradients*maps may include the influence of a confounding factor on the model decision, for example, patient age is often a confounding factor for neurodegeneration.Q. Zhao, Adeli, Pfefferbaum, et al. (2019)modified*Vanilla Gradients*to remove the influence of age as a confounder from sensitivity maps computed from a CNN classifier for Human Immunodeficiency Virus (HIV). Let${\text{f}}_{j}=\left(f_{j1},f_{j2},...,f_{jN}\right)$be the$j^{th}$feature from the final convolutional layer for allNsubjects,$s=\left(s_{1},...,s_{N}\right)$be the CNN score, and$a=\left(a_{1},...,a_{N}\right)$be the subject age. Then, the linear model${\text{f}}_{j}=\beta_{0}+\beta_{1}\text{s}+\beta_{2}\text{a}$was fitted and if$\beta_{2}$was non-zero, then age was defined as a confounder for the$j^{th}$feature. When calculating the*Vanilla Gradients*map, gradients were computed for unconfounded features only. The confounder-free sensitivity maps showed the posterior ventricle was most influenced by age rather than HIV.

#### 5.2.4 Brain age regression

*SmoothGrad*was applied to a CNN trained on a$T_{1}$-weighted brain MRI dataset to predict subject age, and a population-average sensitivity map was computed (Levakov et al., 2020). The ventricles and subarachnoid cisterns were predominantly highlighted in the sensitivity map, which may be related to brain atrophy from the aging process.

#### 5.2.5 Cognitive task decoding

McClure et al. (2023)trained a CNN on activation maps from task-based fMRI to classify the fMRI task and employed*Vanilla Gradients*for model interpretation. To address the shattered gradients limitation of*Vanilla Gradients*the authors utilised adversarial training. More specifically, non-targeted adversarial noise was learned and added to each image, optimised as the smallest magnitude noise that minimised the probability of the correct class. In this way, coupling*Vanilla Gradients*with adversarial training was found to produce gradient maps that were more class discriminative than*Vanilla Gradients*,*Grad*×*Input*, and*SmoothGrad*. However, the maps were still only found to be weakly correlated with class-specific features.

Long short-term memory (LSTM) is a deep learning architecture that is well suited to time series fMRI data because it is designed to process sequence data. However, one limitation of applying*Vanilla Gradients*to LSTM models is the issue of vanishing gradients (Bengio et al., 1994) when backpropagating through many timesteps. Consequently, only features in the latest time steps are highlighted in gradient maps.Ismail et al. (2019)proposed incorporating an attention mechanism into an LSTM, to bypass backpropagating through multiple timesteps during*Vanilla Gradients*. The attention-based LSTM was trained on task-based fMRI to classify the fMRI task, and*Vanilla Gradients*was then able to highlight features in early time steps.

### 5.3 Applications of backpropagation-based methods ( Table 5 )

**Table 5.** Articles using backpropagation-based methods.

Reference	Data	Modality	#Subjects	Method
**AD classification**				
Böhle et al. (2019)	ADNI	sMRI (3D)	344	LRP
Eitel et al. (2019)	ADNI	sMRI (3D)	344	Guided backprop + LRP
**Sex classification**				
Kan et al. (2020)	HCP	sMRI (3D)	1,113	Guided backprop
**Cognitive task decoding**				
Thomas et al. (2019)	HCP	fMRI (ts)	100	LRP

Alzheimer’s Disease Neuroimaging Initiative; HCP, Human Connectome Project; LRP, Layer-wise Relevance Propagation; sMRI, structural Magnetic Resonance Imaging; fMRI, functional magnetic resonance imaging; ts, time series.

#### 5.3.1 Alzheimer’s disease classification

*LRP*and*Guided Backpropagation*were utilised for AD classification after training on ADNI structural MRI data (Böhle et al., 2019;Eitel et al., 2019). In one study, LRP heatmaps were shown to be more class-discriminative than*Guided Backpropagation*maps (Böhle et al., 2019). In a similar approach to theEitel et al. (2019)study, the heatmap analysis was improved by three summary statistics: sum of relevance, mean relevance (to account for brain region size) and relevance gain compared to CN (to find regions where explanations between AD and CN differ the most). All three studies consistently identified the hippocampi and other structures in the temporal lobe as important for AD classification.

#### 5.3.2 Cognitive task decoding

*LRP*has been coupled with a deep learning model trained on task-based fMRI data to predict one of four cognitive states associated with viewing four image categories (body, face, place, or tool) during the task (Thomas et al., 2019). The population-level explanation for each cognitive state was compared against a meta-analysis associated with the keyword from NeuroSynth. The explanations generally matched with the meta-analysis for body and face cognitive states, but less so for place and tool.

### 5.4 Applications of Class Activation Maps ( Table 6 )

**Table 6.** Articles using CAM interpretable methods.

Reference	Data	Modality	#Subjects	Method
**AD classification**				
Azcona et al. (2020)	ADNI	Surface mesh (3D)	435	Grad-CAM
Khan et al. (2019)	ADNI	sMRI (2D)	150	CAM
Tang et al 2019 ^†^	In-house	Histology (2D)	33	Guided Grad-CAM
C. Yang et al. (2018)	ADNI	sMRI (3D)	103	CAM + Grad-CAM
Zhang et al. (2021)	ADNI	sMRI (3D)	1,407	Grad-CAM
**PD classification**				
Williamson et al. (2022)	PPMI	SPECT (3D)	600	Grad-CAM
**ASD classification**				
Li et al. (2020)	In-house	fMRI (ts)	118	Activation maps
**Tumour classification**				
Windisch et al. (2020)	Various ^‡^	sMRI (2D)	2, 479	Grad-CAM
**Tumour segmentation**				
Natekar et al. (2020)	BraTS	sMRI (3D)	461	Grad-CAM
**ICH classification**				
H. Lee et al. (2019)	In-house	CT (2D)	904	CAM
**Sex classification**				
Gao et al. (2019)	Various ^††^	sMRI (3D)	6,008	CAM
Kan et al. (2020)	HCP	sMRI (3D)	1,113	Grad-CAM
Kim and Ye (2020)	HCP	fMRI (ts)	1,094	CAM + Grad-CAM
**Cognitive score prediction**				
W. Hu et al. (2021)	PNC	fMRI (ts)	854	Guided Grad-CAM
Qu et al. (2021)	PNC	fMRI (ts)	800	Grad-RAM

†= Aβplaque morphology classification. ‡= IXI, CyberKnife, TCGA. ††= HCP, FCP, GSP, NKI-RS, CoRR, SLIM.

AD, Alzheimer’s Disease; ADNI, Alzheimer’s Disease Neuroimaging Initiative; ASD, Autism Spectrum Disorder; BraTS, Brain Tumor Segmentation challenge; CAM, Class Activation Map; CoRR, Consortium for Reliability and Reproducibility; CT, Computed Tomography; FCP, Functional Connectome Project; fMRI, functional Magnetic Resonance Imaging; GSP, Brain Genomics Superstruct Project; HCP, Human Connectome Project; ICH, Intracerebral Hemorrhage; IXI, Information Extraction from Images; NKI-RS, Nathan Kline Institute-Rockland Sample; PD, Parkinson’s Disease; PNC, Philadelphia Neurodevelopmental Cohort; PPMI, Parkinson’s Progressive Markers Initiative database; RAM, Recurrent Activation Map; fMRI, functional Magnetic Resonance Imaging; SLIM, Southwest University Longitudinal Imaging Multimodal; sMRI, structural Magnetic Resonance Imaging; SPECT, Single Photon Emission Computed Tomography; TCGA, The Cancer Genome Atlas; ts, time series.

#### 5.4.1 Neurodegenerative disease classification

*CAM*and*Grad-CAM*have been applied to AD and mild cognitive impairment (MCI) classification using a ResNet for 2D MRI (Khan et al., 2019), VGG, and ResNet for 3D MRI (C. Yang et al., 2018;Zhang et al., 2021), and graph convolutional network (GCN) for surface meshes of the cortex and sub-cortical structures (Azcona et al., 2020), all trained on ADNI structural MRI data. Additionally,Tang et al. (2019)applied*Guided Grad-CAM*to amyloid-beta (Aβ) plaque-stained immunohistochemical data to classify plaque morphology, since Aβplaques are a histopathological hallmark of AD.

In their study of AD classification using a VGG,Zhang et al. (2021)showed that applying*Grad-CAM*to lower convolutional layers produced more detailed explanations. However, as lower layers tend to respond to edges/junctions of the brain images, so did the corresponding*Grad-CAM*maps.

A related application of network interpretation is to use it to diagnose failure cases, for example,Khan et al. (2019)evaluated*CAM*for a case of failed classification. In this case, the network attended to structures that are not associated with AD classification, such as the skull. Similarly,Williamson et al. (2022)identified failure cases using*Grad-CAM*for PD classification of SPECT scans, where the presence of noise artifacts and hyperintensities was shown to influence the network decision.

#### 5.4.2 Intracerebral hemorrhage classification

*Grad-CAM*generated explanations for a DL model that detected and classified intracerebral hemorrhage (ICH) sub-types from Computed Tomography (CT) scans of the head (H. Lee et al., 2019). Ground-truth data were available to validate the explanations; the proportion of “bleeding points”, selected by neuroradiologists to indicate the centre of haemorrhagic lesions, overlapping*Grad-CAM*heatmaps was 78%.

#### 5.4.3 Brain tumour classification

Windisch et al. (2020)used*Grad-CAM*to explain tumour classification from structural and diffusion MRI data. As in theKhan et al. (2019)study, results were visually evaluated for cases of correct and incorrect classification. The network focused on the tumour when correctly classifying the scans, while there was no clear attention pattern when the classification failed.

#### 5.4.4 Autism spectrum disorder classification

Li et al. (2020)proposed visualisation of activation maps from a GCN trained to predict ASD from task-based fMRI data. Using an approach analogous to*CAM*but for GCNs, the 25% of graph nodes (representing 21 brain regions) with the highest activation scores after the final graph-convolutional layer were visualised to interpret model classification. The method highlighted the dorsal striatum, thalamus, and frontal gyrus, regions thought to be affected by ASD.

#### 5.4.5 Sex classification

The Dense-CAM network proposed byGao et al. (2019)employed*CAM*in the final layer of a DenseNet (Huang et al., 2017) trained for sex classification, and found the cerebellum to be the most important brain region. On the other hand,Kim and Ye (2020)applied*Grad-CAM*to a GCN trained on rs-fMRI data and found regions involved in the default mode network to be important for sex classification, but not the cerebellum.

#### 5.4.6 Cognitive score prediction

In two studies,*Grad-CAM*was applied to visualise important brain regions for predicting the Wide Range Achievement Test (WRAT) score of healthy individuals from the Philadelphia Neurodevelopmental Cohort (PNC), using a regression (Qu et al., 2021) and classification approach (W. Hu et al., 2021). In one study, a GCN was trained on task-based fMRI data; then,*Grad-CAM*adapted for regression was computed (Qu et al., 2021). Alternatively, subjects were classified into low, medium, and high WRAT score, and*Guided Grad-CAM*was used to identify important brain regions for WRAT score classification (W. Hu et al., 2021). Both studies identified regions of the occipital lobe as important, which is involved in object recognition.

### 5.5 Applications of weight analysis ( Table 7 )

**Table 7.** Articles using weight analysis interpretable methods.

Reference	Data	Modality	#Subjects	Method
**ASD classification**				
Dvornek et al. (2019)	ABIDE	fMRI (ts)	527	Community detection
Li et al. (2021)	Biopoint	fMRI (ts)	115	Community detection
**Tumour segmentation**				
Kori et al. (2020)	BraTS	sMRI (3D)	300	Concept Graphs
Natekar et al. (2020)	BraTS	sMRI (2D)	461	Network Dissection
**Cognitive task decoding**				
Li et al. (2021)	HCP	fMRI (ts)	237	Community detection

ABIDE, Autism Brain Imaging Data Exchange; ASD, Autism Spectrum Disorder; BraTS, Brain Tumor Segmentation challenge; fMRI, functional Magnetic Resonance Imaging; HCP, Human Connectome Project; sMRI, structural Magnetic Resonance Imaging.

#### 5.5.1 Tumour segmentation

The*Concept Graphs*framework was applied to a U-Net brain tumor segmentation model trained on the Brain Tumor Segmentation (BraTS) dataset (Bakas et al., 2017). The method identified multiple concepts at various model layers, such as the whole tumour, tumour core boundaries, and the tumour core region. Concept detection was also used for interpretability of a U-Net tumour segmentation model inNatekar et al. (2020)using*Network Dissection*. Results showed individual filters learned interpretable concepts, including grey and white matter and edema, and separate filters for the whole tumour and the tumour core. These results showed that segmentation networks exhibit modularity in the inference process that can be understood by humans. InKori et al. (2020), in collaboration with a radiologist, inference trails that represent the trail of information in the network were also analysed. The network was shown to take a hierarchical approach to segmentation, starting with the detection of edges at lower layers and moving to the detection of the tumour in upper layers.

#### 5.5.2 ASD classification

Dvornek et al. (2019)incorporated*Community Detection*within their DL model trained for ASD classification on rs-fMRI data. The weights$W\in {\mathbb{R}}^{N\times K}$for*Community Detection*, where$w_{nk}\in W$represents the strength of the connection between brain regionnand communityk, were learned as part of the model. Clustering was then performed for each$k^{th}$community vector$\left[w_{1k},w_{2k},...,w_{Nk}\right]$to assign each brain region as belonging versus not belonging to communityk. Finally, the importance of communitykfor ASD classification was defined as the sum of absolute weights of allk-indexed nodes in the classification model. The three most important communities included brain regions associated with language and social processing, memory, and reward-processing and decision-making.

#### 5.5.3 Cognitive task decoding

GCNs were re-designed for*Community Detection*in BrainGNN (Li et al., 2021). Let$W_{i}^{\left(l\right)}$denote the learnable weights associated with nodeiin graph convolutional layerlof a GNN, where nodeirepresents a fixed brain region with one-hot location encoding$n_{i}$. The authors proposed to encode brain region location in$W_{i}^{\left(l\right)}$by training an MLP on the brain region location$n_{i}$:

$$vec\left(W_{i}^{\left(l\right)}\right)={\text{Θ}}_{2}^{\left(l\right)}\text{ReLU}\left({\text{Θ}}_{1}^{\left(l\right)}n_{i}\right)+b^{\left(l\right)}$$ (12)

where${\text{Θ}}_{1}^{\left(l\right)}$,${\text{Θ}}_{2}^{\left(l\right)}$and$b^{\left(l\right)}$are MLP parameters. The elements${\left(\alpha_{nk}\right)}^{+}$of$\text{ReLU}\left({\text{Θ}}_{1}^{\left(l\right)}\right)\in {\mathbb{R}}^{N\times K}$were interpreted as the non-negative community detection scores of brain regionnbelonging to communityk. In this study, BrainGNN was trained on the biopoint task-based fMRI dataset for ASD classification, as well as on the Human Connectome Project (HCP) task-based fMRI data to classify seven cognitive tasks.

### 5.6 Applications of disentangled latent spaces ( Table 8 )

**Table 8.** Articles using disentangled latent space methods.

Reference	Data	Modality	#Subjects	Method
**Image generation**				
Mouches et al. (2021)	In-house	sMRI (3D)	2,681	Factor: age
	+IXI			
Ouyang et al. (2022)	ADNI	sMRI (3D)	632	Factor: healthy+ disease
Q. Zhao, Adeli, Honnorat, et al. (2019)	In-house	sMRI (3D)	245	Factor: age
F. Zhao et al. (2023)	Various ^‡^	Surface mesh (3D)	2,542	Factor: clinical site
Zuo et al. (2021)	Various ^†^	sMRI (2D)	100	Factor: clinical site
**Tumour classification**				
Afshar et al. (2018)	In-house	sMRI (2D)	233	Capsule network
**Brain age regression**				
D. Hu et al. (2020)	BCP	sMRI+ fMRI (3D)	178	Factor: modality
**Neurodegenerative disease classification**				
T. Wang et al. (2023)	ADNI	sMRI+ PET (3D)	4,126	Factor: modality
	+PPMI	DTI (3D)		

Datasets: †= IXI+ OASIS+ BLSA, ‡= In-house+ BCP+ IBIS.

ADNI, Alzheimer’s Disease Neuroimaging Initiative; BCP, UNC/UMN Baby Connectome Project; BLSA, Baltimore Longitudinal Study of Aging; BraTS, Brain Tumor Segmentation Challenge; fMRI, Functional Magnetic Resonance Imaging; IBIS, Infant Brain Imaging Study; IXI, Information Extraction from Images; OASIS, Open Access Series of Imaging Studies; sMRI, Structural Magnetic Resonance Imaging.

#### 5.6.1 Tumour classification

A*Capsule Network*was trained to classify tumour type (meningioma, pituitary, glioma) from segmented brain MRIs (Afshar et al., 2018). The*Capsule Network*learned to reconstruct input images, where the latent space was constrained to three activation vectors representing the three tumour types. The activation vectors were inspected by perturbing individual vector elements and visualising the reconstructed images, which revealed the*Capsule Network*had learned interpretable tumour features such as tumour size and elongation.

#### 5.6.2 Image generation

One application of latent space disentanglement is training an AE where a latent factor represents age, enabling the generation of MRIs for different ages (Mouches et al., 2021;Q. Zhao, Adeli, Honnorat, et al., 2019). One study jointly trained a supervised age regression network and a VAE where both shared convolutional layers (Q. Zhao, Adeli, Honnorat, et al., 2019). The latent space of the VAE was trained to approximate a prior distribution$p(z|\text{​}\hat{y})$conditioned on the age$\hat{y}$predicted by the regressor. In another study, an AE was coupled with a linear function such that the first parameter of the latent space predicted subject age (Mouches et al., 2021). Age-specific MRI images were synthesised in both studies by adjusting the age-related latent factor.

One study synthesised T1w MRI images at different ages for healthy controls and for patients with AD, through disentangling the effect of AD from healthy ageing on MRIs during image reconstruction (Ouyang et al., 2022). Disentanglement was achieved by learning two orthogonal directions in the latent space of an AE and designing loss functions that encouraged the difference between two successive MRIs of a subject, as represented in the latent space, to be equal to the sum of two components in the healthy and diseased directions. Formally, let$z_{1}$and$z_{2}$be the latent representations of two MRI images acquired for a subject at times$t_{1}$and$t_{2}$, respectively, with$t_{2}>t_{1}$. Then, the vector$\Delta \;z=\left(z_{2}-z_{1}\right)/\left(t_{2}-t_{1}\right)$was constrained by the loss so that$\Delta \;z\approx \text{​}\Delta \;z_{a}+\text{​}\Delta \;z_{d}$, where$\Delta \;z_{a}$and$\Delta \;z_{d}$are parallel to the healthy and disease directions, respectively. The disentangled latent factors were visualised and the AE had learned distinct trajectories for CN, progressive mild cognitive impairment (pMCI), stable mild cognitive impairment (sMCI) and AD subjects.

Another application is harmonisation of MRI across different clinical sites, where the contrast of an MRI image acquired at site A is transformed to appear as if it were acquired at site B, while leaving the subject anatomy unaltered. Disentangled latent spaces have been employed in MRI harmonisation models to learn separate features for anatomy versus image contrast (F. Zhao et al., 2023;Zuo et al., 2021). For example,Zuo et al. (2021)trained a model named CALAMITI for site harmonisation across 10 clinical sites. Two separate encoders,$E_{anat}$and$E_{cont}$, learned feature representations for anatomical and contrast information during image reconstruction, respectively$\theta_{anat}$and$\theta_{cont}$.$E_{anat}$learned anatomical-only features by encouraging$\theta_{anat}$from both T1-w and T2-w images of the same subject and slice number to be identical, thus capturing the shared anatomical features and ignoring contrast. Simultaneously,$E_{cont}$was trained on different slice numbers of the same T1-w and T2-w images to represent the residual features of contrast in$\theta_{cont}$, after accounting for anatomy in$\theta_{anat}$. Similarly,F. Zhao et al. (2023)learned a disentangled VAE latent space consisting of site-related versus non site-related features. ForMsites, a vector of lengthMin the latent space was optimised to represent site-specific features by feeding it into a site classification network and minimising the cross-entropy loss. Both studies visualised the latent space and demonstrated subjects were clustered by site.

#### 5.6.3 Brain age regression

Disentanglement of a network latent space may be advantageous for networks trained on multi-modal data, to decouple modality-specific from shared latent features (D. Hu et al., 2020). Such an approach was adopted when predicting infant brain age from both fMRI and structural magnetic resonance imaging (sMRI) features, where an AE was trained on each modality such that the latent space was divided into modality-specific features and common features (D. Hu et al., 2020). The common feature vectors from both AEs for the same subject were encouraged to be identical by adding an$L_{2}$loss and adversarial loss. Furthermore, each decoder was required to reconstruct the input data from its own common feature vector as well as that from the AE of the other modality, a method known as cross reconstruction. The common feature vector and each modality-specific feature vector were passed to an age prediction network to predict age. Visualisation of the learned latent space demonstrated the latent features were ordered by age.

#### 5.6.4 Neurodegenerative disease classification

Similarly,T. Wang et al. (2023)learned a disentangled latent space to partition features by modality, as well as learn features shared between modalities, when learning from multimodal data. Two AEs were trained, one on each modality, on sMRI/fluorodeoxyglucose positron emission tomography (FDG-PET) for AD classification from ADNI, as well as on sMRI/diffusion tensor imaging (DTI) for PD classification from Parkinson’s Progression Markers Initiative (PPMI). Each latent space was split into modality-specific and common features; a distance loss, defined as the$L_{2}$loss between common features divided by the$L_{2}$loss between modality-specific features, encouraged common features to be identical and modality-specific features to be dissimilar. In addition, cross-reconstruction was adopted, where each decoder generated images using common features learned from both AEs. Disentanglement enabled important brain regions to be identified as sMRI-specific, FDG-PET (or DTI in the case of PD classification)-specific or common to both imaging modalities.

### 5.7 Applications of interpretable hybrid models ( Table 9 )

**Table 9.** Articles using interpretable hybrid models or interpretable intermediate features.

Reference	Data	Modality	#Subjects	Method
**AD classification**				
Abuhmed et al. (2021)	ADNI	sMRI (3D)	1,371	Cognitive scores
		PET (3D)		
E. Lee et al. (2019)	ADNI	sMRI (3D)	801	Disease
				probability map
L. Y.-F. Liu et al. (2020)	ADNI	sMRI (3D)	3,021	Int. features only
Mohammadjafari et al. (2021)	ADNI	sMRI (2D)	408	Prototypes
	+OASIS			
Mulyadi et al. (2023)	ADNI	sMRI (3D)	2,285	Prototypes
	+In-house			
Nguyen et al. (2022)	Various ^†^	sMRI (3D)	2,036	Disease grade map
Qiu et al. (2020)	Various ^††^	sMRI (3D)	1,446	Disease
				probability map
Wolf et al. (2023)	ADNI	PET (3D)	1,245	Prototypes
**ASD classification**				
E. Kang et al. (2022)	ABIDE	fMRI (3D)	985	Prototypes
**Brain age regression**				
Hesse et al. (2023)	IXI	sMRI (2D)	562	Prototypes
**Cognitive/ clinical score regression**				
Shimona D’Souza et al. (2020)	HCP	fMRI (3D)	150	FBNs
	+KKI	DTI (3D)		
**ADHD classification**				
Qiang et al. (2020)	ADHD-200	fMRI (3D)	541	FBNs

† = ADNI+ AIBL+ OASIS+ MIRIAD+ NIFD, †† = ADNI + AIBL + FHS + NACC.

ABIDE, Autism Brain Imaging Data Exchange; AD, Alzheimer Disease; ADHD, Attention Deficit Hyperactivity Disorder; ADNI, Alzheimer’s Disease Neuroimaging Initiative; AIBL, Australian Imaging Biomarker and Lifestyle Flagship Study of Ageing; ASD, Autism Spectrum Disorder; DTI, Diffusion Tensor Imaging; FBN, Functional Brain Network, FHS, Framingham Heart Study; fMRI, functional Magnetic Resonance Imaging; HCP, Human Connectome Project; IXI, Information eXtraction from Images; KKI, Kennedy Krieger Institute; MIRIAD, Minimal Interval Resonance Imaging in Alzheimer’s Disease; NACC, National Alzheimer’s Coordinating Center; NIFD, Frontotemporal lobar Degeneration Neuroimaging Initiative; OASIS, Open Access Series of Imaging Studies; PET, Positron Emission Tomography; sMRI, structural Magnetic Resonance Imaging.

#### 5.7.1 Neurodegenerative disease classification

One blueprint for interpretable intermediate features for AD classification is a heatmap of predicted probabilities of AD across brain regions.Qiu et al. (2020)adopted this approach when designing an NN + NN hybrid model, where the first component was a patch-based CNN that output probability of AD across brain patches. After training, predicted probabilities for 200 voxels were concatenated with non-imaging features (age, gender, mini-mental state examination (MMSE)) and used to train a multi-layer perceptron to predict AD (summarised inFig. 9). However, the heatmaps were less precise and therefore more difficult to interpret than the next two studies discussed. Similarly, an NN+ ML hybrid model learned intermediate probability heatmaps for AD (E. Lee et al., 2019). For the first component, an ensemble of NN classifiers was trained to predict AD or MCI status for each of 93 atlas-derived brain regions, from which a probability heatmap was constructed. The second component was a linear support vector machine (SVM) trained to predict AD status from the probability heatmap. This study followed the “int. features + feature importance” approach and also considered the weights learned by the linear SVM.Nguyen et al. (2022)adopted a comparable approach where instead of learning a brain heatmap of probabilities, voxels were assigned a grade close to 1 if abnormal and close to -1 if healthy. The NN + NN hybrid model consisted of a set of patch-based U-Nets that generated the grade heatmap, followed by a GCN trained to predict CN versus AD versus Frontotemporal Dementia (FTD). The population-average heatmaps in all three studies were highly class-discriminative and were consistent with known disease pathology, focusing predominantly on the temporal lobe for AD, and the frontal lobe for FTD in the case of the study byNguyen et al. (2022).

Three studies employed*prototypes*to introduce interpretability into AD classification models (Mohammadjafari et al., 2021;Mulyadi et al., 2023;Wolf et al., 2023). ProtoPNet has been trained on two public T1-w MRI datasets, ADNI and Open Access Series of Imaging Studies (OASIS) (Marcus et al., 2007), to classify AD (Mohammadjafari et al., 2021).Wolf et al. (2023)trained a variant of ProtoPNet to predict AD from FDG-PET images from the ADNI database, and two of the prototypes highlighted the ventricles and occipital lobe. In another study, a prototype-based model was trained on T1-w MRI from ADNI and an in-house dataset, and the*prototypes*learned were reconstructed from the latent space to a 3D feature vector [AD diagnosis, MMSE, age] (Mulyadi et al., 2023). Furthermore, prototypical brains for each diagnosis class (CN, progressive MCI, stable MCI and AD) were compared to individual scans; for example, a CN subject differed most from the AD prototypical brain in the amygdalae. However, a limitation of prototype-based explanations is they are low resolution because of upsampling from a latent space to the image space.

Abuhmed et al. (2021)explored yet another hybrid model design and predicted AD clinical scores as interpretable intermediate features. The NN+ ML hybrid model predicted the prognosis of AD status at month 48 from multi-modal data collected at baseline and months 6, 12, and 18. The first component was a Bi-LSTM model trained to forecast seven cognitive scores (such as the MMSE) at month 48. The second component was an interpretable ML classifier trained separately to forecast disease status at month 48 from the seven forecasted cognitive scores, subject age, gender, and education. Taking the “int. features + feature importance” approach, explanations were also generated from the ML classifier; however, the explanations were only in relation to the cognitive scores and not the neuroimaging data.

#### 5.7.2 Autism spectrum disorder classification

A*prototype-based*approach was adopted when classifying ASD from functional connectivity (FC) matrices computed from rs-fMRI data from the ABIDE dataset (E. Kang et al., 2022). The first component of the hybrid model was a transformer network (seeSection 4.2.5) that generated latent features for a subject, and then the predicted class was determined by the class prototype closest to the latent features. To enable interpretability, a decoder was trained to reconstruct the input FC from an individual’s latent features and was also used to decode a more prototypical FC. For example, by subtracting a reconstructed FC of a control subject from the ASD-typical version, the authors found regions such as the right cingulate gyrus and the occipital and frontal poles as the most different from ASD for this individual.

#### 5.7.3 Brain age regression

*Prototypes*were also employed for predicting brain age from T1w MRI images (Ixi dataset, n.d.), as well as predicting gestational age from fetal ultrasound (US) images (Hesse et al., 2023).*Prototypes*were adapted for regression as follows: prototypes were not assigned to a class, but each prototype was replaced with the closest latent representation of a training image, and associated with the corresponding age label. The predicted age is the weighted mean of age labels of all prototypes within a fixed distance from the sample in the latent space. The method was able to display the four prototypical brains most similar to a test image.

#### 5.7.4 Attention deficit hyperactivity disorder classification

Another choice for intermediate interpretable features in a hybrid model is to learn functional brain networks (FBNs) that are important for the DL task.Qiang et al. (2020)trained such an ML + ML hybrid model to classify attention deficit hyperactivity disorder (ADHD) from rs-fMRI data. First, a VAE was trained on fMRI data and the latent representations learned by the VAE were used to learn FBN weights using Lasso regression (penalised regression with L1 penalty). For the second component, FC matrices were constructed from the FBN weights and used to train an ML classifier to predict ADHD. The FBNs learned by the VAE were shown to be similar to those derived from another state-of-the-art method.

#### 5.7.5 Cognitive/clinical score regression

In a similar manner toQiang et al. (2020),Shimona D’Souza et al. (2020)learned FBNs as interpretable intermediate features for cognitive and clinical score prediction. The authors coupled representation learning with an NN that predicted cognitive or clinical scores. Intermediate FBNs were learned from rs-fMRI functional connectivity matrices using structurally-regularised Dynamic Dictionary Learning (sr-DDL). Simultaneously, an LSTM was trained from the subject-specific FBN coefficients. The ML + NN hybrid model was trained to predict the Cognitive Fluid Intelligence Score for healthy subjects from the HCP dataset (Van Essen et al., 2013), as well as clinical scores (Autism Diagnostic Observation Schedule, Social Responsiveness Scale and Praxis) for ASD subjects from the Kennedy Krieger Institute (KKI) dataset (Bigler, 2008). Fifteen learned FBNs for both HCP and KKI data were presented as the model explanations. For example, several of the networks learned to predict the Cognitive Fluid Intelligence Score were involved in the Medial Frontal Network and the Frontal Parietal Network, which play a role in decision-making, attention, and working memory.

### 5.8 Applications of interpretable generative models ( Table 10 )

**Table 10.** Articles using generative models.

Reference	Data	Modality	#Subjects	Method
**AD classification**				
Bass et al. (2020)	ADNI	sMRI (3D)	1,053	Generative additive maps
Bass et al. (2022)	ADNI	sMRI (3D)	1,053	Generative additive maps
Baumgartner et al. (2018)	ADNI	sMRI (3D)	1,288	Generative additive maps
Lanfredi et al. (2020)	ADNI	sMRI (3D)	825	Generative
				deformation fields
Z. Liu et al. (2021)	ADNI	sMRI (3D)	1,344	Generative
				deformation fields
**Brain age regression**				
Bass et al. (2022)	Various ^†^	sMRI (3D)	12,434	Generative additive maps
**Brain tumour and stroke segmentation**				
Bercea et al. (2023)	Various ^‡^	sMRI (2D)	1,412	Generative additive maps
Sanchez et al. (2022)	BraTS	sMRI (2D)	1,251	Generative additive maps
Wolleb et al. (2022)	BraTS	sMRI (2D)	N/A	Generative additive maps

† = UK Biobank + dHCP, ‡ = ATLAS v2.0 + IXI+ FastMRI.

AD, Alzheimer’s Disease; ADNI, Alzheimer’s Disease Neuroimaging Initiative; BraTS, Brain Tumor Segmentation Challenge; dHCP, Developing Human Connectome Project; IXI, Information eXtraction from Image; N/A, Not Available; sMRI, structural Magnetic Resonance Imaging.

#### 5.8.1 Neurodegenerative disease maps

Several studies trained a generative adversarial network (GAN) (Goodfellow et al., 2020) on the ADNI structural MRI dataset to predict disease effect maps for AD, considering either MCI or CN as the control class (Bass et al., 2020,^2022^;Baumgartner et al., 2018;Lanfredi et al., 2020;Z. Liu et al., 2021).Baumgartner et al. (2018)developed a visual attribution method based on a conditional GAN (***VA-GAN***). In this work, an additive mapM(x)was learned as a function of an input imagexfrom the AD class, such that the modified imagex+M(x)appears cognitively normal. In contrast to learning an additive map,Lanfredi et al. (2020)trained a GAN to generate a deformation field, known as deformation field interpretation (***DeFI-GAN***), which was shown to produce sparser disease effect maps than VA-GAN. The deformation field transforms an image from the AD class to the MCI class by modelling brain atrophy. As such, deformation-based approaches are only appropriate for modelling diseases where brain atrophy is the predominant imaging marker. The same deformation field approach was employed byZ. Liu et al. (2021), but using a cycleGAN that generated modified AD and CN images. The Jacobian of the deformation field was visualised as the disease effect map.

The aforementioned methods assume that the category labels of the test data (either real or estimated by a separate classifier) are known during testing, meaning that the models can generate explanations, but cannot perform the classification.Bass et al. (2020)developed a model that both classified disease and generated disease effect maps. By incorporating a classification network, this model obviates the need for previously classified data during testing. A VAE-GAN was trained to disentangle class-relevant features from background features, and therefore to separate the effects of healthy aging from disease. The mean and variance of predicted disease effect maps were sampled from the latent space during testing, as opposed to from a single additive map for each subject. The method was applied to brain structural MRI data from ADNI as inBaumgartner et al. (2018), and disease effect maps were shown to improve when compared to*VA-GAN*and gradient-based methods (shown inFig. 1). In addition to classification, the method was extended for regression of MMSE from structural MRI ADNI data; regression of age from Biobank brain structural MRI scans; and regression of birth age from developing Human Connectome Project (dHCP) data (Bass et al., 2022). All of these studies produced AD disease effect maps that successfully modelled atrophy of the ventricles, hippocampus, and cortical grey matter known to occur in AD.

#### 5.8.2 Brain tumour and stroke segmentation

More recently, state-of-the-art diffusion models (Ho et al., 2020) have been trained to predict disease effect maps (anomaly maps) for neuroimaging datasets (Bercea et al., 2023;Sanchez et al., 2022;Wolleb et al., 2022). Two studies trained a Denoising Diffusion Probabilistic Model (DDPM) on sMRI images from the BraTS dataset to convert a cancerous MRI to appear healthy, and a third study trained a generative model to transform MRI brain images of stroke patients to appear healthy. In all studies, the generated healthy image was subtracted from the original to produce the anomaly map.Wolleb et al. (2022)trained an unconditional DDPM and a classifier, and then used classifier guidance to transform an MRI from cancerous to healthy. In contrast,Sanchez et al. (2022)trained a conditional DDPM and employed classifier-free guidance to alter the cancerous images.Bercea et al. (2023)implemented a two-stage approach, where stroke-effected regions were removed from the image in stage one, and then stage two comprised an in-painting generative model to fill in these erased regions as healthy. The anomaly maps in all three studies were shown to identify pathological brain regions successfully.

### 5.9 Applications of deep structural causal models ( Table 11 )

**Table 11.** Articles using deep structural causal models.

Reference	Data	Modality	#Subjects	Method
**Image generation**				
Pawlowski et al. (2020)	UK Biobank	sMRI (2D)	13,750	DSCM
Rasal et al. (2022)	UK Biobank	Surface meshes (3D)	14,502	DSCM
Reinhold et al. (2021)	In-house	sMRI (2D)	77	DSCM

DSCM, Deep Structural Causal Model; sMRI, structural Magnetic Resonance Imaging.

Reinhold et al. (2021)extended the*DSCM*in Eqn. 5 to model causal effects for structural MRI images from an MS cohort by adding duration of MS symptoms, expanded disability severity score, lesion volume, and image slice number. Counterfactual difference maps were explored, such as the counterfactualdo(l=0 ​ mL)for a brain MRI of an MS patient, where the model successfully removed the MS lesions from the counterfactual image.

Furthermore,Rasal et al. (2022)modified a DSCM to synthesise 3D surface meshes of the brain stem by introducing graph convolutional layers into the VAE. The authors performed interventions on the population-mean brain stem, as well as generating subject-specific counterfactual surface meshes for variables such as age and sex. Realistic counterfactual meshes were generated for scenarios outside the true data distribution, for example,do(age=80year-old)when the maximum participant age was 70 years old.

### 5.10 Applications of attention mechanisms ( Table 12 )

**Table 12.** Articles using attention-based methods.

Reference	Data	Modality	#Subjects	Method
**Image segmentation**				
Gu et al. (2020)	In-house	sMRI (2D)	36	Spatial, channel and
				non-local attention
**Disease Classification**				
Jin et al. (2020)	ADNI	sMRI (3D)	1,832	Spatial attention
	+In-house			
Sarraf et al. (2023)	ADNI	fMRI (ts)	1,744	Self-attention
		+ sMRI (2D)		
M. Zhao et al. (2022)	ABIDE	fMRI (ts)	2,622	Time-axis attention
	+In-house			
**Brain age regression**				
Dahan et al. (2022)	dHCP	Surface meshes (3D)	588	Self-attention

ABIDE, Autism Brain Imaging Data Exchange; ADNI, Alzheimer’s Disease Neuroimaging Initiative; dHCP, developing Human Connectome Project; fMRI, functional Magnetic Resonance Imaging; sMRI, structural Magnetic Resonance Imaging; ts, time series.

#### 5.10.1 Image segmentation

Gu et al. (2020)introduced*channel*,*spatial*, and*non-local attention*blocks in a modified U-Net to improve the performance of medical image segmentation tasks. More specifically, they used*spatial attention*blocks throughout the decoder layers of the U-Net by combining both higher (from the decoder) and lower resolution (from the encoder) feature maps, similar to that proposed previously (Oktay et al., 2018).*Channel attention*blocks were also introduced after each decoding layer by global average pooling and global max pooling (Woo et al., 2018). The latter was also introduced as “scale attention”, which assigns a weight for each of the decoder outputs to enable differential attention to be assigned to a given input. The final*non-local block*was introduced at the lowest resolution level (the bottleneck of the U-Net) due to its complexity. They showed the*spatial attention*maps from the trained network were able to highlight the object to be segmented, suggesting that the use of*attention*enhanced the ability of the network to focus on target areas to facilitate performance.

#### 5.10.2 Disease classification

A 3D*spatial attention*network was used to classify AD using two large structural MRI datasets (ADNI and an in-house database) (Jin et al., 2020). Following grey matter segmentation, volumes were inputted into a 3D-CNN, which contained a*spatial attention*block after the first three convolutional layers to highlight important regions in the feature maps. However, the*spatial attention*module contained an ReLU rather than a sigmoid activation function. Thus, probability values for each spatial location were not produced, but nevertheless, the method was able to identify those brain regions correlated with atrophy, characteristic of AD.

*Attention*has also been introduced into a hybrid DL framework to classify SCZ and ASD using an in-house and the ABIDE rs-fMRI dataset, respectively (M. Zhao et al., 2022). Features were first extracted from the imaging data using principal components analysis, and 50 independent components (IC) were retained per subject, each of which was a times series. An attention-guided convolutional recurrent neural network (C-RNN) was then used to process the IC time series data, and a deep neural network (DNN) for processing functional network connectivity (FNC) matrices. The C-RNN*attention*block aimed to highlight which rs-fMRI-derived ICs were more significant for prediction. The*attention*module was comparable to that proposed byWoo et al. (2018), which uses both max and average pooling layers, butM. Zhao et al. (2022)applied these along the time axis. The outputs of these two separate networks were concatenated and passed through a logistic regressor to obtain the final classification result.

Sarraf et al. (2023)developed an optimised vision transformer, OViTAD, for classifying healthy control (HC), MCI, and AD brains using rs-fMRI and sMRI data. The authors also generated attention maps for AD versus HC versus MCI classification for the different*self-attention*heads, as well as global-level attention maps extracted from the last feature vector.

#### 5.10.3 Brain age regression

Finally,Dahan et al. (2022)introduced the Surface Vision Transformer, which adapted the image transformer model to surface domains. More specifically, surface meshes were transformed into triangular patches, flattened into feature vectors, and then inputted into the transformer model (Touvron et al., 2021). The main task of their proposed study was to perform phenotype regression tasks using cortical surface metrics from the dHCP. The authors also produced average attention maps for either regression of postmenstrual age at scan and gestational age at birth.

## 6 Evaluation of iDL Explanations

Of utmost importance, iDL explanations need to be evaluated for biological validity and robustness. Biological validity refers to whether explanations capture the true, underlying biological or pathological processes, and robustness assesses the stability of an explanation under varying conditions. Other properties of iDL explanations that were evaluated in the literature are continuity, selectivity, and downstream task performance. These properties will be discussed below in the context of the 75 studies included in this review.

### 6.1 Biological validity

A key challenge for iDL in neuroimaging is that only appropriately trained medical specialists, for example, radiologists, can validate explanations. Explanations for natural images can usually be readily validated by a general audience; for example, the model predicts “castle” and the explanation highlights the castle turrets. In contrast, years of specialised medical training are required to identify imaging biomarkers, such as regional brain atrophy in neurodegenerative diseases. Studies may be conducted where clinicians evaluate iDL explanations. However, due to limited clinician availability, quantitative and automated validation metrics are more desirable.

Most of the studies we reviewed did not validate (26 out of 75) or only qualitatively validated (31 out of 75) the iDL explanations—for example, many studies compared salient brain regions identified in the explanations with those previously reported. Several fMRI studies leveraged Neurosynth, a meta-analysis platform that can return functional keywords correlated to iDL explanations, and compared these keywords against the literature.

The remaining 18 studies quantitatively compared iDL explanations to ground-truth which were obtained through various sources (Table 13). A noteworthy example is where longitudinal imaging data were available, such as in the ADNI database. For subjects that progressed from CN/MCI to AD, a ground-truth disease effect map may be computed by subtracting the registered AD image from the CN/MCI image (Bass et al., 2020,^2022^;Baumgartner et al., 2018;Lanfredi et al., 2020). The explanation maps were then quantitatively compared to ground-truth disease effect maps using normalised cross correlation (NCC). Overall, explanations from interpretable generative models achieved substantially higher NCC (Bass et al., 2020,^2022^;Baumgartner et al., 2018) than explanations from popular post-hoc methods, such as*CAM*,*Guided Backpropagation*, and*Integrated Gradients*.

**Table 13.** Quantitative metrics to evaluate biological validity of iDL explanations.

Reference	Ground-truth data source	Metric
**Perturbation-based**		
Y. Liu et al. (2019)	cGAN-based statistics	# brain regions
C. Yang et al. (2018)	8 hold-out subjects	precision-recall curve
**Gradient-based**		
Ismail et al. (2019)	Off-task data	% relevant features on-task
Levakov et al. (2020)	VBM meta-analysis	Mean VBM for top 1% regions
**Backpropagation-based**		
Thomas et al. (2019)	NeuroSynth meta-analysis	Mean F1 score
**Weights analysis**		
Natekar et al. (2020)	Ground-truth segmentation	IoU
**Disentangled latent space**		
Mouches et al. (2021)	Ground-truth segmentation	Lateral ventricle volume
Ouyang et al. (2022)	ADAS-Cog scores	Correlation
**Interpretable hybrid models**		
Qiu et al. (2020)	*Post-mortem* tissue	Correlation
**Generative models**		
Bass et al. (2020)	ADNI disease effect map	NCC
Bass et al. (2022)	ADNI disease effect map	NCC
Baumgartner et al. (2018)	ADNI disease effect map	NCC
Bercea et al. (2023)	Ground-truth segmentation	Dice
Lanfredi et al. (2020)	ADNI disease effect map	NCC
Sanchez et al. (2022)	Ground-truth segmentation	Dice
Wolleb et al. (2022)	Ground-truth segmentation	Dice
**Deep structural causal models**		
Reinhold et al. (2021)	Image segmentation	MS lesion volume
**Attention**		
Jin et al. (2020)	AD MMSE scores	Correlation

AD, Alzheimer’s Disease; ADAS-Cog, Alzheimer’s Disease Assessment Scale – Cognitive Subscale; ADNI, Alzheimer’s Disease Neuroimaging Initiative; cGAN, conditional generative adversarial network; IoU, intersection over union; MMSE, Mini-mental state examination; MS, Multiple Sclerosis; NCC, normalised cross-correlation; VBM, Voxel-based morphometry.

### 6.2 Robustness

Robustness was not evaluated in the majority of studies (62 out of 75). In the remaining studies, the robustness of population-level explanations was considered with respect to different training data (Gao et al., 2019;Jin et al., 2020;Thibeau-Sutre et al., 2020), data pre-processing methods (Li et al., 2019;Mellema et al., 2020), and model and iDL settings (Dvornek et al., 2019;Eitel et al., 2019;Kim & Ye, 2020;Levakov et al., 2020;Li et al., 2018;Shimona D’Souza et al., 2020;Thibeau-Sutre et al., 2020). Three studies compared population-level explanations with the same DL task and model architecture but where the model was trained on different sources of data, and all concluded explanations were stable across datasets. For example,Jin et al. (2020)compared attention maps from a ResNet trained on structural MRI ADNI data versus a similar in-house dataset and found the maps were significantly correlated (r = 0.59). A few studies considered explanations trained on the same data source but with different pre-processing methodologies, investigating different atlases and atlas granularities during registration (Li et al., 2019;Mellema et al., 2020). Furthermore, robustness of explanations across different model and iDL settings was evaluated, including cross-validation folds (Dvornek et al., 2019;Kim & Ye, 2020;Shimona D’Souza et al., 2020;Thibeau-Sutre et al., 2020); parameter initialisation (Eitel et al., 2019;Thibeau-Sutre et al., 2020); hyperparameter values (Li et al., 2018;Thibeau-Sutre et al., 2020); and models within an ensemble (Levakov et al., 2020).

Data preprocessing methods, hyperparameters, and model parameters all influence the explanations produced. Concerning data preprocessing, skull stripping often alters downstream explanations (Druzhinina et al., 2021;Khan et al., 2019). In another example,Mellema et al. (2020)showed the level of atlas granularity during registration altered the important features identified for ASD classification. Regarding hyperparameters, the selection of regularisation weights for*Meaningful Perturbations*changed the explanation masks for AD classification (Thibeau-Sutre et al., 2020). Evidence also suggests that different runs of identically trained, randomly initialised models are associated with markedly different explanations (Eitel et al., 2019;Thibeau-Sutre et al., 2020). It is important to be aware that bias may be present in iDL explanations from sources such as data preprocessing and hyperparameter selection and to assess explanations for such bias.

The robustness of explanations under different conditions may be quantitatively assessed using various similarity measures (seeTable 14). Some studies directly compared explanations using overlap measures such as the Dice coefficient or Hausdorff distance. Other studies initially converted an explanation into a vector of mean values fornatlas-derived brain regions and then compared vectors using correlation (Dvornek et al., 2019;Jin et al., 2020), cosine similarity (Thibeau-Sutre et al., 2020) or percentage agreement between top regions (Eitel et al., 2019).

**Table 14.** Quantitative metrics to evaluate robustness of iDL explanations.

Reference	Robustness across…	Metric
**Perturbation-based**		
Eitel et al. 2019 ^†^	Models (initialisation)	L2-norm
		+relevant region coherence
Li et al. (2018)	Models (hyperparameters)	# important ROIs
Thibeau-Sutre et al. (2020)	Datasets	Cosine similarity
Thibeau-Sutre et al. (2020)	Models (hyperparameters)	Cosine similarity
Thibeau-Sutre et al. (2020)	Models (cv folds + initialisation)	Cosine similarity
**Gradient-based**		
Levakov et al. (2020)	Models (ensemble)	Dice + Hausdorff distance
**Class activation maps**		
Kim and Ye (2020)	Models (cv folds)	Relevant region coherence
**Weights analysis**		
Dvornek et al. (2019)	Models (cv folds)	Correlation + Dice
**Interpretable hybrid models**		
Shimona D’Souza et al. (2020)	Models (cv folds)	Mean inner-product
**Attention**		
Jin et al. (2020)	Datasets	Correlation

†:Eitel et al. (2019)evaluated robustness across multiple method categories, not only perturbation-based.

cv, cross-validation; ROI, Region of Interest.

### 6.3 Other interpretable method properties ( Table 15 )

**Table 15.** Other properties and quantitative metrics for iDL explanations.

Reference	Interpretable method	Metric
**Continuity**		
Nigri et al. (2020)	Perturbation-based	$L_{2}$ norm
**Selectivity**		
Nigri et al. (2020)	Perturbation-based	Correlation
**Downstream task performance (disentangled latent space methods only)**		
Ouyang et al. (2022)	Disentangled latent space	Classification accuracy

#### 6.3.1 Continuity

Similar images should have similar explanations, as originally proposed byMontavon et al. (2018).Nigri et al. (2020)measured continuity by slightly perturbing 50 input images and then calculating the mean$L_{2}$-norm between explanations of the original and perturbed image. The authors compared the continuity of the*Swap Test*and*Occlusion*and found the*Swap Test*to be the superior method.

#### 6.3.2 Selectivity

Regions with the highest relevance in the explanation should result in the largest change in model prediction when removed from the input image (Montavon et al., 2018). For example, sensitivity maps were computed byNigri et al. (2020), highlighting those image regions swapped (*Swap Test*) or occluded (*Occlusion*) that resulted in a large change in model prediction. Reverse sensitivity maps were then generated by removing the complement of each image patch and recording the change in model prediction. Subsequently, Pearson correlations were computed to assess the relationship between the standard and reversed sensitivity maps, with strong negative correlations expected when the property of selectivity is satisfied. Each image from the MS class underwent lesion in-painting, such that MS lesions appeared to be healthy tissue in the MRI image. Explanations were generated for the original and in-painted images, and the difference between their mean values was computed, with larger differences across all images suggesting a more selective iDL method.

#### 6.3.3 Downstream task performance (disentangled latent space methods only)

Performance relates to whether the latent space distinguishes classes sufficiently for a given downstream task. InOuyang et al. (2022), DL models were trained on the disentangled latent embeddings for two classification tasks to understand if the latent space learned a meaningful structure. The evaluation metric was the test set classification accuracy.

## 7 Discussion and Conclusion

In this review, we identified 75 neuroimaging studies that utilised iDL methods, and we classified the methods into five post-hoc and five intrinsic categories. To the best of our knowledge, this is the first systematic review of iDL in neuroimaging with a notably more extensive review of intrinsic methods than found in the literature (Thibeau-Sutre et al., 2023). In addition, we found five properties of iDL explanations that were investigated and are important when considering the suitability of an iDL method for adoption.

The most common iDL methods utilised were*class activation maps*,*perturbation-based*and*gradient-based*methods. Post-hoc methods are popular because they are well established in computer vision tasks, easy to implement, and readily available in DL packages. However, historically, post-hoc methods were designed for and validated on natural images and may be inappropriate for neuroimaging tasks. For example, saliency methods were shown to only focus on a few discriminative features of a class (Bass et al., 2020;Zhang et al., 2021), rather than identifying all imaging features, which may be sub-optimal for diagnosis and treatment. Their reliability is also questionable as some post-hoc methods, in particular*Guided backpropagation*and*Guided Grad-CAM*still produce convincing explanations despite randomised model weights or data labels (Adebayo et al., 2018). In contrast, intrinsic methods are generally more appropriate for neuroimaging because they are designed specifically for the application, for example, constructing a causal graph specific to MS (Reinhold et al., 2021). Additionally, generative models produced explanations with substantially higher correlation to ground-truth disease markers compared to explanations from several post-hoc methods (Bass et al., 2020,^2022^;Baumgartner et al., 2018). Nevertheless, intrinsic interpretable deep learning is still an emerging field, and such methods are currently more time-consuming to implement than post-hoc methods.

We will now provide some recommendations for researchers when using iDL with neuroimaging datasets. First, we suggest utilising multiple iDL methods, including several across different post-hoc method categories (such as*Occlusion*,*LRP*, and*GradCAM*) and one intrinsic method that is best suited for the project application, end-user requirements, objectives, etc. It is important to carefully select one intrinsic method during the design phase as it can be time-consuming to implement. For pre-existing models, incorporating an appropriate attention mechanism and retraining the model may be feasible. Then, compare explanations from different methods and prioritise features highlighted across all methods.

Second, recall that various confounding factors, such as data preprocessing, random initialisation, and cross-validation, can affect explanations. Therefore, we advise averaging explanations across cross-validation folds and multiple runs to improve robustness. Also, consider visualising explanations for a reasonable selection of model preprocessing and hyperparameter settings. If using multiple neuroimaging datasets, we recommend adopting a standardised pre-processing pipeline to reduce the risk of biased explanations.

Third, validating explanations across an entire test dataset is crucial rather than limiting assessments to a select few samples. This comprehensive validation helps ensure the generalisability of the explanations. Consider acquiring ground truth to validate explanations quantitatively, such as computing disease affect maps from longitudinal imaging datasets. If not possible, then impartially compare explanations to existing physiopathological literature. In summary, do not unquestioningly trust the explanations produced by an iDL method.

When applying iDL methods for neuroimaging, an important concern is the complexity of the biological mechanisms underlying the data and the interactions between multiple imaging features. Many interpretability methods identified in this review do not consider the causal mechanisms that contribute to the data nor the impact of confounding factors in the explanations. We have, however, discussed state-of-the-art causal models that attempt to address causality in interpretability, and we foresee such models playing an important role in the future of iDL (Pawlowski et al., 2020;Reinhold et al., 2021). We also conclude a suite of standardised, quantitative evaluation metrics to compare performance across iDL methods needs to be established to promote the trustworthiness of iDL methods.

## Data Availability

The images used in some of the figures are owned by the third-party organisation Alzheimer’s Disease Neuroimaging Initiative (ADNI) and are publicly available athttp://adni.loni.usc.edu/data-samples/access-data/. Please find the ADNI protocol and ethics statement athttp://adni.loni.usc.edu/wp-content/themes/freshnews-dev-v2/documents/clinical/ADNI-2_Protocol.pdf.
